# Evolutionary and Characteristic Analysis of RING-DUF1117 E3 Ubiquitin Ligase Genes in *Gossypium* Discerning the Role of GhRDUF4D in *Verticillium dahliae* Resistance

**DOI:** 10.3390/biom11081145

**Published:** 2021-08-03

**Authors:** Yan-Peng Zhao, Jian-Ling Shen, Wen-Jie Li, Na Wu, Chen Chen, Yu-Xia Hou

**Affiliations:** 1 Zhengzhou Research Base, State Key Laboratory of Cotton Biology, School of Agricultural Sciences, Zhengzhou University, Zhengzhou 450001, China; ypzhao@zzu.edu.cn (Y.-P.Z.); shenjianling8@163.com (J.-L.S.); 13781949770@163.com (W.-J.L.); w15093389925@163.com (N.W.); C18337103690@163.com (C.C.); 2 State Key Laboratory of Cotton Biology, Institute of Cotton Research, Chinese Academy of Agricultural Sciences, Anyang 455000, China; 3 College of Science, China Agricultural University, Beijing 100193, China

**Keywords:** E3 ubiquitin ligase, DUF1117, upland cotton, GhRDUF4D, *Verticillium dahliae*

## Abstract

*Verticillium* wilt, primarily induced by the soil-borne fungus *Verticillium dahliae*, is a serious threat to cotton fiber production. There are a large number of *really interesting new gene* (RING) domain-containing E3 ubiquitin ligases in Arabidopsis, of which three (At2g39720 (AtRHC2A), At3g46620 (AtRDUF1), and At5g59550 (AtRDUF2)) have a domain of unknown function (DUF) 1117 domain in their C-terminal regions. This study aimed to detect and characterize the RDUF members in cotton, to gain an insight into their roles in cotton’s adaptation to environmental stressors. In this study, a total of 6, 7, 14, and 14 *RDUF* (*RING-DUF1117*) genes were detected in *Gossypium arboretum*, *G. raimondii*, *G. hirsutum*, and *G. barbadense*, respectively. These *RDUF* genes were classified into three groups. The genes in each group were highly conserved based on gene structure and domain analysis. Gene duplication analysis revealed that segmental duplication occurred during cotton evolution. Expression analysis revealed that the *GhRDUF* genes were widely expressed during cotton growth and under abiotic stresses. Many *cis*-elements related to hormone response and environment stressors were identified in GhRDUF promoters. The predicted target miRNAs and transcription factors implied that *GhRDUFs* might be regulated by gra-miR482c, as well as by transcription factors, including MYB, C2H2, and Dof. The *GhRDUF* genes responded to cold, drought, and salt stress and were sensitive to jasmonic acid, salicylic acid, and ethylene signals. Meanwhile, *GhRDUF4D* expression levels were enhanced after *V. dahliae* infection. Subsequently, *GhRDUF4D* was verified by overexpression in *Arabidopsis* and virus-induced gene silencing treatment in upland cotton. We observed that *V*. *dahliae* resistance was significantly enhanced in transgenic *Arabidopsis*, and weakened in *GhRDUF4D* silenced plants. This study conducted a comprehensive analysis of the *RDUF* genes in *Gossypium*, hereby providing basic information for further functional studies.

## 1. Introduction

Cotton (*Gossypium*) is well-known as the most important natural fiber. The cultivated tetraploids upland cotton (*Gossypium hirsutum*, AD_1_) and sea island cotton (*G. barbadense*, AD_2_) have resulted from hybridization and genome doubling from the diploid ancestors of *G. herbaceum* (A_1_) or *G. arboreum* (A_2_) and *G. raimondii* (D_5_) [1,2,3]. More than 90% of cultivated cotton crops comprise upland cotton, owing to its high fiber production volumes and high tolerance to various environmental conditions. *Verticillium* wilt is primarily caused by the soil-borne fungus *V. dahliae*, and is a serious disease that poses a significant threat to the fiber industry. Because of cotton’s limited germplasm diversity, *V. dahliae*-resistant cotton varieties are difficult to obtain via traditional breeding practices [4]. Efforts have been made to determine the molecular basis for *Verticillium* wilt tolerance in cotton. A set of *V. dahliae*-responsive genes have been identified that primarily involve the jasmonic acid (JA), salicylic acid (SA), and ethylene (ET) signaling pathways [5,6,7,8]. Moreover, the function of several individual genes, including *GhPMEI3* [9], *GhLAC15* [10], *GhGhARPL18A-6* [11], *GbSOBIR* [12], and *GhGPA* [13], have been investigated in cotton for protection from *Verticillium* wilt. These studies have contributed to our understanding of the complex innate defense mechanisms against *V. dahliae* infection in cotton.

The ubiquitin-26S proteasome system plays a major role in protein degradation, an important regulatory mechanism that mediates cell responses to intracellular signals and adaptation to environmental conditions [14,15]. The ubiquitination cascade is catalyzed by the ubiquitin-activating enzyme (E1), ubiquitin-conjugating enzyme (E2), and ubiquitin protein ligase (E3) [16,17,18]. In *Arabidopsis thaliana*, there are 2 E1 enzymes, 37 E2 enzymes, and more than 1400 predicted E3 ubiquitin ligases [19]. The large number of E3 ligases is primarily responsible for substrate specificity [14]. The E3 ligases can be grouped into four defined classes, according to the presence of HECT (homologous to E6-associated protein carboxyl terminus), U-box, really interesting new gene (RING), or cullin–RING ligases [14,20,21]. The RING domain is a cysteine (Cys)-rich region containing 40–60 amino acids. It spatially contains eight conserved Cys and histidine (His) residues as metal ligands, which can chelate two zinc ions to transfer ubiquitin to target proteins [19]. In addition, the RING domains were found to be essential for catalyzing the E3 ligase activity of RING-containing proteins [22].

RING domains are divided into two canonical types, C3HC4 (RING-HC) and C3H2C3 (RING-H2), in terms of a Cys or His amino acid residue at the metal ligand position five [23,24]. In addition, other modified types of RING domains containing E3 ligases have been found in *A. thaliana*, *Oryza sativa*, *Ostreococus tauri*, and *Brassica rapa* [19,25,26,27,28]. The majority of RING-containing proteins have been actively examined in in vitro ubiquitination assays [25]. The biological function of RING-containing proteins is to participate in anther development, secretory pathways, plant defense, and pathogen response [25,27]. For example, SDIR1, a RING-type E3 ligase, modulates salt and drought stress responses in *Arabidopsis* [29,30]. Furthermore, a study has shown that the rice C3HC4 protein improves resistance to *Pseudomonas syringae* pv. *tomato* DC3000 in transgenic *A. thaliana* [31]. The reduced expression of the rice E3 enzyme XB3 compromised the avirulent breed of *Xanthomonas oryzae* pv *oryzae* resistance [32]. OsRFPH2-10, a RING-H2 finger E3 ubiquitin ligase, is involved in antiviral defense in the early stages of rice dwarf virus infection [33].

A large number of RING domain-containing E3 ubiquitin ligases have been identified in *Arabidopsis* [25]. Among them, At3g46620 (AtRDUF1), At5g59550 (AtRDUF2), and At2g39720 (AtRHC2A) have a domain of unknown function (DUF) 1117 domain. AtRDUF1, AtRDUF2, and AtRHC2A are RING-H2 type RING finger family proteins [25]. *AtRDUF1* has been reported to positively regulate salt stress [34], whereas the suppression of *AtRDUF1* and *AtRDUF2* reduces tolerance to abscisic acid (ABA)-mediated drought stress in *Arabidopsis* [35]. Furthermore, *AtRDUF1* and *AtRDUF2* respond to chitin, a plant defense elicitor, with 7.9- and 9.0-fold increased expression 30 min after induction, respectively [36]. These studies indicate that the RDUF genes are involved in biotic and abiotic plant adaptations to the environment. To investigate the potential role of the RDUF genes in *Gossypium*, we characterized the RDUF gene family in four cotton species: *G. arboretum*, *G. raimondii*, *G. hirsutum*, and *G. barbadense*. In addition, we investigated *GhRDUF4D* ectopic expression in *Arabidopsis* and the virus-induced gene silencing (VIGS) approach in upland cotton. This study sheds light on *GhRDUF4D*’s role against *V. dahliae* infection in cotton, and will help in understanding the function of *RDUF* genes in plant immunity.

## 2. Materials and Methods

### 2.1. Characterization of RDUF Gene Members in Gossypium

The genome datasets of *G. hirsutum* acc. TM-1 (AD_1_, ZJU_v2.1) [2], *G. barbadense* acc. Hai7124 (AD_2_, ZJU_v1.1) [2], and its assumed ancestors of *G. arboreum* Shixiya 1 (A_2_, CRI_v1.0) [37] and *G. raimondii* (D_5_, JGI_v2.1) [38] were downloaded from the CottonGen website [39]. The Basic Local Alignment Search Tool (BLAST) program BLASTp was used to detect candidate *RDUF* genes using the *Arabidopsis* RDUF amino acid (aa) sequences of AtRDUF1 (AT3G46620), AtRDUF2 (AT5G59550), and AtRHC2A (AT2G39720) as queries. Then, the Pfam RDUF1117 domain (PF06547) was used for the examination of RDUF members via a HMMER search. The *RDUF* genes were named based on their chromosomal location. Genes in tetraploid cotton were named as per the homologous relationship of each subgenome, with “A” and “D” indicating the homologous genes in At and Dt subgenomes, respectively. In addition, the theoretical molecular weight (MW) and isoelectric point (pI) were estimated by ExPASy [40], and the subcellular localization was evaluated by TargetP-2.0 Server (available online: http://www.cbs.dtu.dk/services/TargetP/ accessed on 30 December 2020). To detect *cis*-elements in the RDUF promoter regions, the 2 kb upstream regions of the start codon of the *RDUF* genes were submitted to the PlantCARE database [41].

### 2.2. Phylogenetic, Gene Structure and Conserved Domain, and Motif Analysis

A maximum likelihood (ML) phylogenetic tree was built using the bootstrap test method with 1000 replicates [42], and then the tree was visualized via the Interactive Tree of Life (iTOL) online tool [43]. Subsequently, gene structure and the conserved domain of the RDUF proteins were detected and displayed using the Gene Structure Display Server (GSDS, v2.0) [44], SMART [45], and TBtools (v1.0983) [46] softwares. Moreover, the conserved motifs in the RDUF proteins were identified using the MEME program [47], and the functional annotations were confirmed using the InterProScan website.

### 2.3. Chromosomal Location, Gene Synteny, and RNA-Seq Dataset Analysis

The chromosomal localization of the *RDUF* genes were determined and displayed using the Mapchart 2.2 software [48], based on the downloaded genomic datasets. MCScanX [49] software was used for gene synteny analysis, whereas Circos (v0.69) [50] software was used for graphical depiction. The expression profiles of *GhRDUF*s were analyzed from publicly released RNA-Seq datasets in silico, with the BioProject accession number PRJNA490626 [2]. Transcript levels were calculated with HISAT and StringTie softwares [51], in the form of fragments per kilobase million (FPKM) values.

### 2.4. Transcription Factors (TFs) and miRNAs in Targeting GhRDUF Genes

TFs involved in the regulation of the *GhRDUF* genes were predicted using PlantRegMap [52]. The cDNA of the *GhRDUF* homologs were submitted to the psRNATarget website to search for potential miRNAs [53]. Then, Cytoscape (v3.7.2) was used to display the regulatory relationships between the predicted TFs, miRNAs, and the targeted *GhRDUF* genes [54].

### 2.5. Cotton Seedlings under Hormone Treatments

*G. hirsutum* cv. Zhongzhimian No. 2 were stored in our laboratory and used for gene expression analysis in response to plant hormone treatments. The cotton seedlings were cultivated in Hoagland liquid medium. The seedlings were sprayed uniformly at the three-leaf stage with 2 mM methyl salicylic acid (MeSA), 100 μM methyl jasmonate (MeJA), or ethylene (ET) released from 5 mM ethephon. The leaves from three cotton seedlings were collected at each time points of 0, 3, 6, 12, 24, and 48 h after spraying with MeSA, MeJA, and ET, respectively, and then immediately frozen in liquid nitrogen for total RNA extraction.

### 2.6. GFP Vector Construction and Fluorescent Visualization

The full-length cDNA of *GhRDUF4D* without the termination codon was cloned into the pCAMBIA2300-GFP vector, via the ClonExpress^®^ MultiS One Step Cloning Kit (Vazyme, Nanjing, China). The precursor sequence (gra-miR482c) and its mutant (gra-miR482c-mut) was biosynthesized by BGI Biotechnology Co., Ltd. (Wuhan, China), and then inserted into the pBI121 vector. After transformation into the *A. tumefaciens* strain GV3101, the transient expression assay was performed on tobacco leaves using an MMA solution (10 mM MgCl_2_, 10 mM N-morpholino ethanesulfonic acid and 200 mM acetosyringone), to validate the interactional relationships between gra-miR482c and *GhRDUF4D* using an in vivo plant imaging system (NightSHADE LB 985, Berthold, Germany).

### 2.7. Overexpression Vector Construction and Screening of Transgenic Arabidopsis

The full cDNA length of *GhRDUF4D* was cloned to confirm its sequence. The ClonExpress^®^ MultiS One Step Cloning Kit (Vazyme, Nanjing, China) was used to construct *GhRDUF4D* to the pCAMBIA2300 vector under the control of a CaMV35S promoter, the recombinant vector was named as 35S::*GhRDUF4D* in the present study. The 35S::*GhRDUF4D* construction was introduced into the *A. tumefaciens* strain GV3101, and then transformed into *A. thaliana* using the floral-dip method. Positive transgenic *Arabidopsis* were confirmed using the reverse transcription polymerase chain reaction (PCR) methods. The specific primers used in this study are provided in Supplementary Table S1. The T_0_–T_3_ seeds were screened in MS medium containing antibiotics. The T_3_ transgenic *Arabidopsis* lines with the correct segregation ratio were used for the *V. dahliae* resistance analysis.

### 2.8. VIGS Vector Construction and Assays Performing

Based on the expression module, *GhRDUF4D* was selected for functional analysis using the virus-induced gene silencing (VIGS) approach. In brief, the 300 bp fragment of *GhRDUF4D* was cloned from the root tissue of *G. hirsutum* cv. Zhongzhimian No. 2. Then, ClonExpress^®^ MultiS One Step Cloning Kits (Vazyme, Nanjing, China) were used to link the tobacco rattle virus (TRV) vector (pYL156) with the *Xba* I and *Bam*H I restriction sites, the recombined vector was named as TRV::*GhRDUF4D* in this study. Next, the recombined vector TRV::*GhRDUF4D*, the pYL156 empty vector TRV::00, the marker vector TRV::*CLA1* (*Cloroplastos alterados 1*), and the auxiliary vector pLY192 were introduced into the *A. tumefaciens* strain GV3101. Subsequently, a single colony of the strain was resuspended in an MMA solution to an OD600 of 1.0. Finally, the suspension of TRV::00 and TRV::*GhRDUF4D* were infected onto the cotyledons of the cotton seedlings. When the photobleaching phenotype was presented in the marker construct of TRV::*CLA1*, the true leaves were collected from each treatment to detect *GhRDUF4D* expression using RT-qPCR. Subsequently, the *GhRDUF4D* silenced plants and the control were subjected to *V. dahliae* inoculation.

### 2.9. V. dahliae Infection and Disease Evaluation

The highly aggressive, defoliating *V. dahliae* strain, Vd991, was used for inoculation. In brief, the spore suspensions of Vd991 were prepared at concentrations of 1 × 10^7^ spore mL^−1^ with sterile water. The cotton and *Arabidopsis* plant roots were then incubated with the conidial suspension for 10 min. The roots of Arabidopsis seedlings planted on MS medium were treated with 2 μL of 5 × 10^3^ spore mL^−1^ conidial suspension for infection. The disease index test was calculated as previously described [5]. Cotton stems were cut from each line at the same position, using a microscope to determine vascular wilt symptoms. The fungal biomass was detected according to the method described by Liu et al. [55]. In brief, total DNA was extracted from cotton seedling stems at 3 weeks post-inoculation, and then the specific primers were employed to detect fungal DNA using quantitative PCR (Table S1). The *V. dahliae* recovery assay was performed as per Zhang et al. [11]. The significant differences for disease index and fungal biomass analysis were determined using the Student’s *t* test.

### 2.10. Reactive Oxygen Species (ROS) Analysis and Callose

ROS was detected using 3,3′-diaminobenzidine (DAB) staining. Leaves from TRV::0 and TRV::*GhRDUF4D* cotton plants from 12 h after inoculation were suspended in DAB staining solution (1 mg/mL, pH = 7.5) in darkness for 8 h. Then, leaves were decolorized in 95% ethanol and absolute ethanol in boiling water, until the green color faded. Next, 70% glycerin was added to soak the leaves; they were then observed under a stereomicroscope. At least three plants were sampled for each treatment.

Callose depositions were observed using aniline blue staining. Leaf samples at 7 days post-inoculation (dpi) were first destained in a fixative solution (3:1 ethanol/acetic acid) for 3 h, and then soaked in 70% and 50% ethanol for 2 h. Afterwards, the leaves were transferred into water for 12 h and destained in NaOH solution (10%, *w*/*v*) for 2 h. Finally, the leaves were stained with 0.01% (*w*/*v*) aniline blue for 3 h, and visualized using fluorescence microscopy.

### 2.11. RT-qPCR Analysis

Total RNA was isolated using RNA extraction kits (Vazyme, Nanjing, China). cDNA was reverse-transcribed using the PrimeScript™ RT reagent kit (TaKaRa, Dalian, China), as per the manufacturer’s instructions. Specific primers were designed via the Primer6 software and are listed in Table S1. RT-qPCR analysis was performed as per Zhao et al. [56]. The housekeeping genes, *GhUBQ7* in cotton and *AtSAND* in *Arabidopsis*, were used as internal references. The relative expression levels were calculated using the 2^−^^ΔΔCT^ method. The assays were performed in triplicate. The significant differences were determined using the Student’s *t* test.

## 3. Results

### 3.1. Identification and Phylogenetic Analysis of RDUF Family Genes

**Figure 1:**
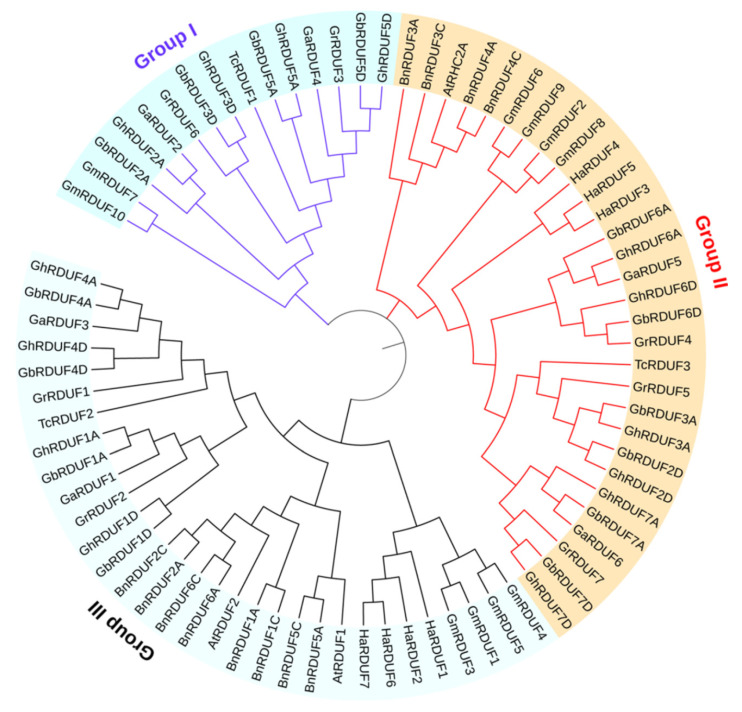
Phylogenetic analysis of the RDUF protein family. At, *Arabidopsis thaliana*; Bn, Brassica napus; Ga, G. arboretum; Gr, G. raimondii; Gh, G. hirsutum; Gb, G. barbadense; Gm, Glycine max; Ha, Helianthus annuus; Tc, Theobroma cacao.

The Pfam family of DUF1117 (PF06547) was used as a query to search for RDUF. At the same time, the AtRDUF amino acid (aa) sequences of AtRDUF1 (AT3G46620), AtRDUF2 (AT5G59550), and AtRHC2A (AT2G39720) in *Arabidopsis* were used for the BLASTp search in the *Gossypium* protein database. We detected 6, 7, 14, and 14 *RDUF* genes in the A_2_, D_5_, AD_1_, and AD_2_ genomes, respectively. We observed that the *RDUF* gene number in the tetraploid *G. hirsutum* and *G. barbadense* was probably twice than that observed in the diploid cottons (*G. arboreum* and *G. raimondii*), suggesting hybridization and whole genome duplication (WGD) events. Meanwhile, 3 *TcRDUF* genes in cacao (*Theobroma cacao*), 10 *GmRDUF* genes in soybean (*Glycine max*), 12 *BnRDUF* genes in oilseed (*Brassica napus*), and 7 *HaRDUF* genes in sunflower (*Helianthus annuus*) were also detected. The phylogenetic tree showed that the 76 RDUF proteins were categorized into 3 groups: Group I, Group II, and Group III (Figure 1). In addition, we also found that the RDUF family proteins were clustered closely in the different species, indicating that RDUF proteins evolved independently after separation from other species. Another phylogenetic tree containing monocotyledonous crop RDUFs was built, including three OsRDUF proteins from *Oryza sativa*, five ZmRDUF proteins from *Zea mays*, and eight TaRDUF proteins from *Triticum aestivum* (Table S2). The RDUFs in monocotyledonous crops were clustered far from those found in dicotyledonous crops (Figure S1, in green clade), suggesting that the RDUFs in monocotyledonous and dicotyledonous crops likely evolved independently. The RDUF-protein phylogenetic trees from the four cotton species were also analyzed. We found that the RDUF members in all four species were categorized into three groups (Figure S2).

Data from the 41 *RDUFs* in *Gossypium* were also investigated. The coding sequence (CDS) length of the 41 *RDUF* genes ranged from 953 bp (*GrRDUF4*) to 1312 bp (*GbRDUF7A*). Most of the *RDUF* genes did not contain an intron, but *GaRDUF3*, *GrRDUF4*, *GbRDUF2D*, and *GbRDUF7A* had two exons each. The amino acid sequences of the RDUF proteins were also characterized. The MW ranged from 34.13 kDa (GbRDUF3A) to 46.92 kDa (GbRDUF7A), and the pI ranged from 5.17 (GbRDUF4A) to 9.27 (GaRDUF4). Subcellular location analysis showed that 23 of the 41 RDUF proteins were predicted to be located in the nucleus, and 13 RDUF proteins were predicted to be located in the chloroplasts (Table 1).

### 3.2. Chromosomal Localization and Gene Synteny Analysis of RDUF Genes

To reveal the homolog relationships between the *RDUF* genes in *Gossypium*, gene localization on chromosomes as well as gene synteny analysis were performed. The results showed that the *RDUF* genes in diploid cotton species were distributed on six chromosomes, with two *GrRDUF* genes localized on chr12 in *G. raimondii*. In tetraploid cotton species, we observed that seven *RDUF* genes of the At subgenome were distributed on seven chromosomes, with each *RDUF* gene located on one chromosome. The seven *RDUF* genes of the Dt subgenome were located on six chromosomes, with *RDUF2D* and *RDUF3D* located on the D04 chromosome (Figure S3). Gene localization analysis revealed that most of the *RDUF* loci were highly symmetric between the At and Dt subgenomes. *RDUF* gene localization in the At subgenomic chromosomes consistently agreed with their homologs in the Dt subgenomic chromosomes. Nevertheless, *RDUF2A* and *RDUF3A* were located on the A03 and A04 chromosomes, respectively, but their homologs, *RDUF2D* and *RDUF3D*, were all located on the D04 chromosome.

**Figure 2:**
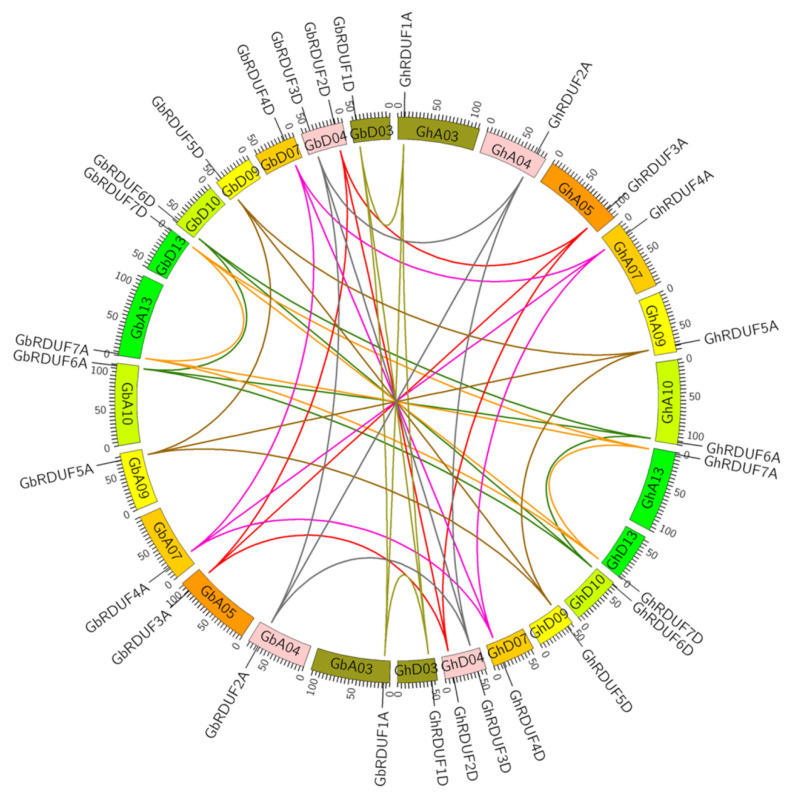
Synteny relationship between *RDUF* genes in *G. hirsutum* and *G. barbadense*. The relationship is presented using Circos software. The homologous chromosomes in At and Dt subgenomes are displayed in the same color. The synteny relationships between the *RDUF* genes are identified by different colors. Bottle green lines, ortholog or paralog genes of *RDUF6A*/*D*; light brown lines, ortholog or paralog genes of *RDUF1A*/*D*; dark brown lines, ortholog or paralog genes of *RDUF5A*/*D*; red lines, ortholog or paralog genes of *RDUF3A*/*2D*; orange lines, ortholog or paralog genes of *RDUF7A/D*; pink lines, ortholog or paralog genes of *RDUF4A*/*D*; and gray lines, ortholog or paralog genes of *RDUF2A/3D*.

Gene synteny analysis was performed to understand the relationships of the *RDUF* genes between *G. hirsutum* and *G. barbadense*. We observed that homologous genes in or between each cotton genome were highly syntenic. The *RDUF* genes in *G. hirsutum* were consistent with their orthologs in *G. barbadense* (Figure 2). We also investigated whether *GhRDUF1A/D* and *GhRDUF4A/D*; *GhRDFU2A/3D* and *GhRDUF5A/D*; and *GhRDUF3A/2D*, *GhRDUF6A/D* and *GhRDUF7A/D* might be duplicated genes (Figure S4). The *RDUF* genes in the diploid cotton species (*G. arboreum* and *G. raimondii*) were twice more than those in cacao, reflecting that at least one WGD event occurred after cotton diverged from cacao. To explore the different selective constrains on the *RDUF* genes, the Ka/Ks ratios for the duplicated genes were calculated (Table S3). The majority of the Ka/Ks values of the *RDUF* gene pairs were <1, suggesting that the *RDUF* genes primarily experienced purified selection. Furthermore, 3 *RDUF* gene pairs exhibited neutral selection, whereas 13 *RDUF* duplicate gene pairs might have experienced positive selection in the 4 cotton species.

### 3.3. Conserved Structure and Domains in GhRDUF Proteins

**Figure 3:**
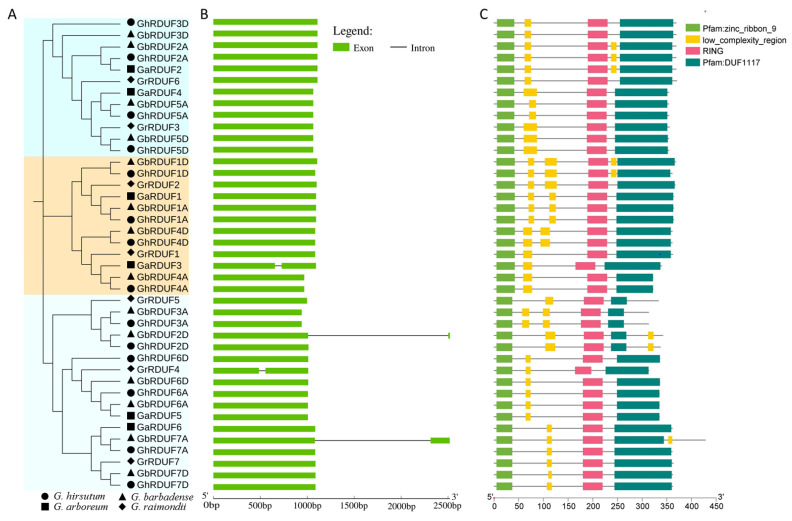
Gene structure and conserved domains in RDUF members in *Gossypium*. (**A**) Phylogenetic tree of RDUF proteins in four cotton species. (**B**) Gene structure of exons and introns in *RDUF* genes. (**C**) The conserved domains in RDUF proteins.

A total of 41 RDUF proteins were used to understand phylogenetic relationships and gene structures. They were classified into three groups, in agreement with those in each cotton species (Figure 3A). The RDUF proteins in the A genome and At subgenome, as well as their orthologs in the D genome and the Dt subgenome tended to consistently form one clade, indicating that the tetraploid cottons might have originated from a hybridization and WGD event of the two diploid cottons. Gene structure analysis displayed that most of the *RDUF* genes contained only one exon (Figure 3B). Consistently, RDUF members with similar structures were grouped in the same clade.

The conserved domains were analyzed to understand the function of RDUF proteins in cotton. All the RDUF proteins in the four cotton species contained the zinc_ribbon_9 domain at the C-terminal, the RDUF117 domain at the N-terminal, the RING domain, and a low_complexity_region domain. Characteristically, the GrRDUF5, GbRDUF3A, GhRDUF3A, GbRDUF2D, and GhRDUF2D proteins contained an abbreviated RDUF117 domain. GbRDUF2D and GhRDUF2D contained the low_complexity_region domain at the N-terminal. GbRDUF2A, GhRDUF2A, GaRDUF2, GhRDUF1D, and GbRDUF1D possessed the low_complexity_region domain between the RING and the RDUF117 domain (Figure 3C). Subsequently, the conserved motifs were detected using the MEME website. All 41 RDUF proteins contained motif_3, motif_5, motif_6, and motif_7. Most of the RDUF proteins contained motif 2, except for GaRDUF3. GrRDUF4 did not contain motif 1, whereas the RDUF3A/2D clade was without motif_4. GrRDUF4 and GrRDUF7 did not have motif_8, and the RDUF1A/D clade was without motif_9. However, only the RDUF6A/D clade had motif_10 (Figure S5). The motif functions were annotated in the Pfam software, with motif_1 belonging to the RING finger domain, motif_3 and motif_6 belonging to the zinc-ribbon domain, and motif_4 belonging to the RDUF1117 domain (Table S4). In addition, the amino acid sequences of the 14 GhRDUF proteins and their homolog alignments were recorded using the Clustalx software. These results showed that the sequences of the conserved domains were highly similar. The conserved metal ligand positions and zinc (Zn^2+^) coordinating amino acids were also investigated. We observed that all 14 GhRDUF proteins contained a His at metal ligand positions four and five, suggesting that the GhRDUF proteins belong to the RING-H2 type (Figure S6).

### 3.4. Cis-Elements in the GhRDUFs Promoter and the Targeting TFs

**Figure 4:**
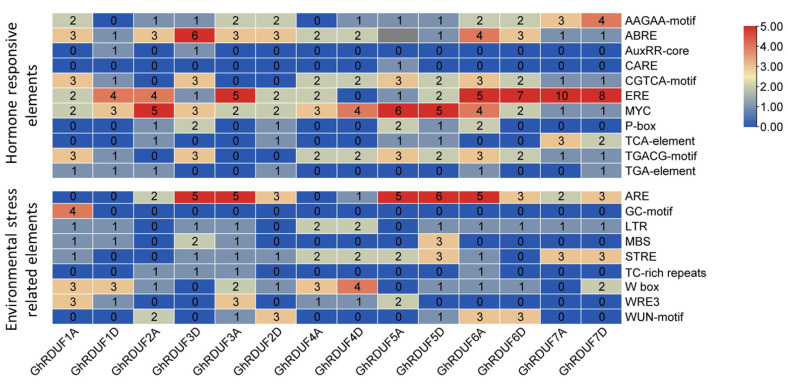
*Cis*-elements in GhRDUF promoter regions. Hormone response elements: AAGAA-motif, abscisic acid responsive element; ABRE, abscisic acid responsiveness element; CGTCA-motif, MeJA-responsiveness *cis*-acting regulatory element; AuxRR-core, auxin responsiveness *cis*-acting regulatory element; MYC, MeJA-responsive *cis*-acting regulatory element; ERE, ethylene responsive element; P-box, gibberellin-responsive element; TGACG-motif, MeJA-responsiveness element; TCA-element, salicylic acid responsiveness element; TGA-element, auxin-responsive element. Environmental stress-related elements: GC-motif, anoxic specific inducibility enhancer-like element; ARE, anaerobic induction element; LTR, low-temperature responsiveness element; MBS, MYB binding site involved in drought-inducibility; TC-rich repeats, defense and stress responsiveness *cis*-element; STRE, stress response element; WUN-motif, wound-responsive element; W-box, plant defense signaling *cis*-acting element; and WRE3, wound inducibility.

Globally, over 90% of cotton crops are upland cotton, owing to its higher fiber production and environmental adaptation. The promoters of the 14 *GhRDUF* genes were submitted in the PlantCARE database to investigate the *cis*-elements. In total, 75 *cis*-elements were predicted to exist in the GhRDUF promoter regions, with more *cis*-elements implicated in the light response, including Box 4, G-Box, and MRE elements (Table S5). The participation of *cis*-elements in the environmental and hormone stress responses are highlighted in Figure 4. Among the 11 hormone response elements predicted in the GhRDUF promoters, ERE (*cis*-acting ethylene responsive element), MYC (*cis*-acting element involved in MeJA stress), and ABRE (*cis*-acting element involved in abscisic acid response) elements were the most abundant, indicating that the *GhRDUF* genes may primarily respond to ET, MeJA, and ABA stress. Nine environmental stress-related elements were identified. The majority of them were involved in anaerobic induction (ARE), plant defense signaling (W-box), and stress responses (STRE). In particular, more W-box *cis*-elements were found in the GhRDUF4D promoter region, implying that *GhRDUF4D* participates in cotton’s response to disease.

*Cis*-elements bind TFs to regulate the precise initiation of gene transcription. Thus, the targeting TFs of *GhRDUFs* were predicted using the PlantRegMap server, with a total of 188 relationships identified, including 35 TF families and 14 *GhRDUF* genes (Figure S7). It appears that *GhRDUF1* homologous genes are regulated by additional TFs, such as those of ERF, Dof, and MYB. Meanwhile, many *GhRDUF* genes were regulated by the stress TFs of MYB, C2H2, and Dof, indicating that *GhRDUF* might regulate STREs.

### 3.5. GhRDUF Targeting miRNAs Predict the Regulatory Network of GhRDUF4D and gra-miR482c

**Figure 5:**
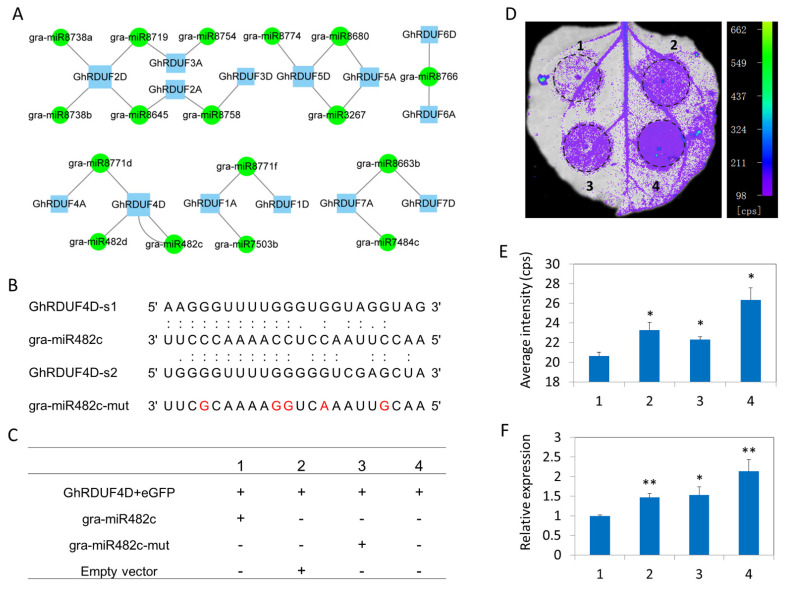
miRNAs targeting the *GhRDUF* genes, and the regulatory network of *GhRDUF4D* and gra-miR482c. (**A**) Network of miRNAs and the target *GhRDUFs*. The predicted regulation miRNAs are shown on a green background in circles, the target *GhRDUF* genes are marked with blue rectangles. The regulation and targeting level are shown with varying degrees. (**B**) The base-pairing interaction between gra-miR482c and *GhRDUF4D*, and the sequence of mutant gra-miR482c. The five point mutations in gra-miR482c-mut are marked in red. (**C**) Vector groups in transient expression assays for each lane. (**D**) Co-infiltrated tobacco leaves photographed under ultraviolet light. The same areas marked by circular dotted lines were used for fluorescence intensity and gene expression analyses. (**E**) The average fluorescence intensity in each lane. (**F**) Relative expression levels of *GFP* in each lane. Mean ± standard error (SE), *n* = 3. “*” and “**” indicate that the data were significantly different at the *p* values of <0.05 and <0.01, respectively.

miRNAs have been widely investigated for their role in the regulation of plant development, as well as in the abiotic and biotic stress responses. In this study, 17 putative miRNAs targeting 14 *GhRDUF* genes were predicted by the psRNATarget website, including 27 interaction relationships. We observed that *GhRDUF2D* was the most targeted, and that it interacted with four miRNAs. Most of the homologous *GhRDUF* genes were regulated by the same miRNAs, indicating that they have similar functions. We highlighted that *GhRDUF4D* is possibly regulated simultaneously by gra-miR482c and gra-miR482d. Furthermore, gra-miR482c regulated *GhRDUF4D* expression with two binding sites, implying a strong regulatory relationship (Figure 5A). The regulatory network between *GhRDUF4D* and gra-miR482c was thus examined using a transient transformation assay. The five point mutations (gra-miR482c-mut) of gra-miR482 were used as the control (Figure 5B). Different vector groups were infiltrated in tobacco leaves and photographed under an ultraviolet light 3 days after infiltration (Figure 5C). We observed that the average fluorescent intensity in the *GhRDUF4D*-*GFP* and gra-miR482c precursor group was significantly impaired, compared with the control (Figure 5D,E). Moreover, the coexpression of the *GhRDUF4D-GFP* and gra-miR482c precursors generated an apparent reduction in the expression level of *GFP* (Figure 5F). These results indicated that gra-miR482c mediates the degradation of *GhRDUF4D*.

### 3.6. Expression Profiling of GhRDUF Genes in Upland Cotton

**Figure 6:**
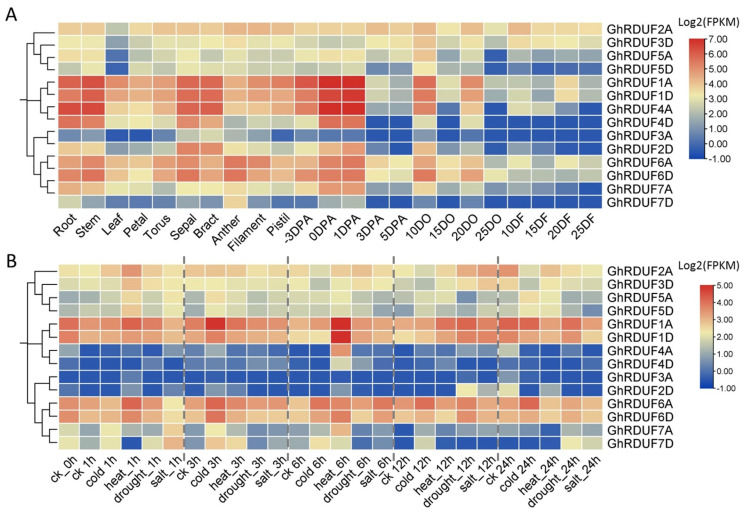
Expression patterns of *GhRDUF* genes in upland cotton development and under abiotic stresses. (**A**) Expression profiles of *GhRDUF* genes in cotton tissues and during development. DO and DF indicate the DPA ovule and fiber, respectively. (**B**) Expression profiles of *GhRDUF* genes under abiotic stresses. FPKM in log2 scale was used for heat map visualization.

Gene expression models are important for gene function analysis. The public expression datasets in upland cotton were used for gene expression analysis, including the different tissues, ovule, and fiber development stages [2]. Most of the homologous genes showed similar expression patterns. *GhRDUF3A* and *GhRDUF7D* genes were barely expressed in upland cotton, whereas *GhRDUF1A/D*, *GhRDUF4A/D*, and *GhRDUF6A/D* homologous genes were abundantly expressed in the root, stem, sepal, bract, early developing ovules (−3, 0, and 1 DPA), and the 10 and 20 DPA ovules. These results indicate that these genes are involved in cotton reproduction and vegetable development. Meanwhile, *GhRDUF1A/D* was preferentially expressed in 20 DPA fibers, suggesting its role in the secondary wall thickening of fiber (Figure 6A).

The expression profiles of the *GhRDUF* genes under abiotic stress were also investigated, including in cold, heat, drought, and salt conditions. Extensive expression changes were detected under these stresses. The expression of the *GhRDUF2A/3D* genes was induced after 1 h of heat stress, and at 12 h under drought and salt stress. There was an impaired expression after 6, 12, and 24 h of cold stress. *GhRDUF5A/D* genes were upregulated at 1 h of heat and cold stress, at 3 and 12 h of cold stress, and at 24 h of cold and heat stress. *GhRDUF1A/D* genes were significantly elevated after 3 h of cold stress, and at 6 h under heat stress. In particular, *GhRDUF4A/D* genes were downregulated at 6 h under heat stress. *GhRDUF6A/D* genes responded to cold stress at 3, 6, 12, and 24 h. *GhRDUF7A/D* genes were abundantly expressed at 1, 3, 6, and 12 h when exposed to cold conditions (Figure 6B). The expression patterns of *GhRDUF* genes under abiotic stress were consistent with the many environment response elements that were predicted in the promoter region (Figure 4C).

**Figure 7:**
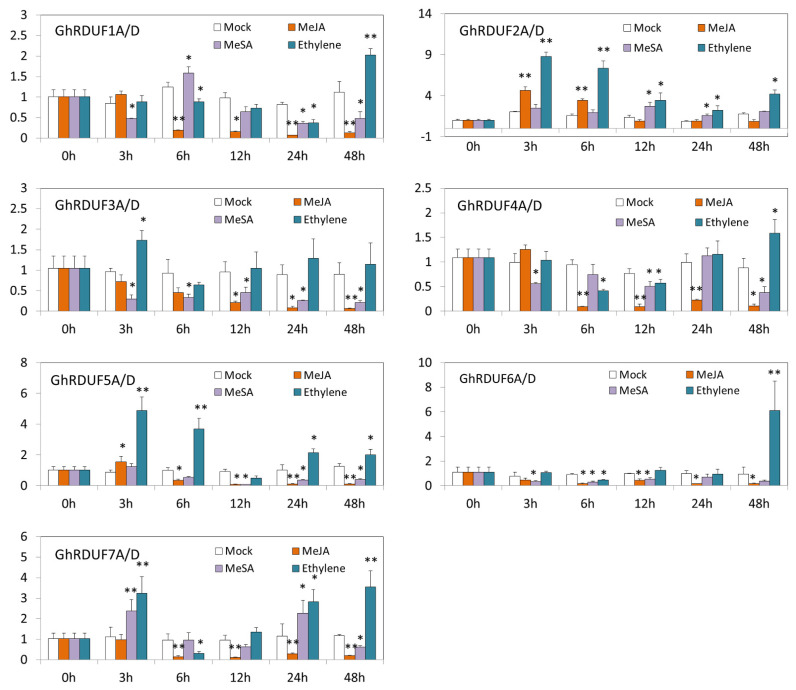
Expression profiles of *GhRDUF* genes under plant hormone treatments. Mean ± SE, *n* = 3. “*” and “**” indicate that the data were significantly different at the *p* values of <0.05 and <0.01, respectively.

Plant hormone signals play a pivotal role in plant autoimmunity, particularly MeJA, MeSA, and ethylene. The response of the *GhRDUF* genes to these three hormones was investigated in this study. We observed that the highest expression of the *GhRDUF* genes was induced under the ethylene treatment (Figure 7). The transcript levels of *GhRDUF1A/D*, *GhRDUF4A/D*, and *GhRDUF6A/D* were upregulated 48 h after ethylene spraying. Many *GhRDUF* genes had impaired expression when exposed to the MeJA and MeSA conditions. For example, the expression levels of *GhRDUF1A/D*, *GhRDUF3A/D*, *GhRDUF4A/D*, and *GhRDUF6A/D* were weakened under the MeJA and MeSA treatments. Other *GhRDUF* genes showed intricate expression patterns. For example, *GhRDUF2A/D* was elevated under MeJA and MeSA treatments at many time points, *GhRDUF5A/D* was abundantly expressed under MeJA treatment at 3 h, and *GhRDUF7A/D* was expressed under MeSA treatment at 3 and 24 h.

### 3.7. Overexpression of GhRDUF4D Enhanced the V. dahliae Resistance in Arabidopsis

**Figure 8:**
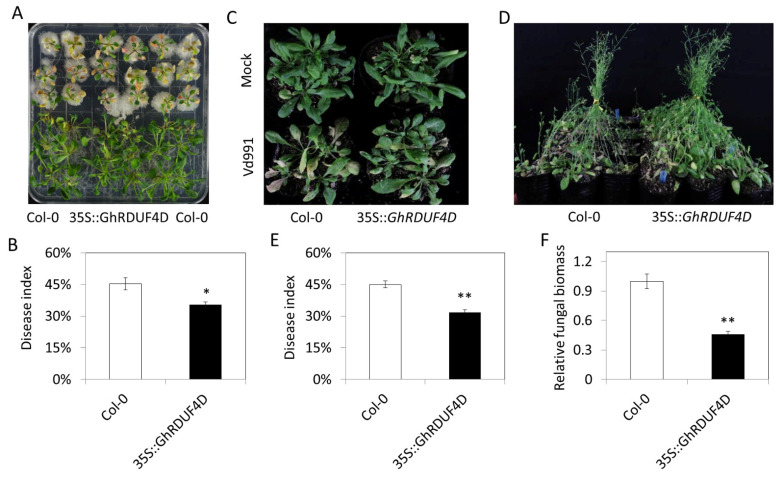
Overexpression of *GhRDUF4D* conferred *V. dahliae* resistance in Arabidopsis. (**A**) Col-0 and OE::*GhRDUF4D* plants inoculated with Vd991 in MS medium plate; (**B**) statistical analysis of disease index in plants in MS medium; (**C**,**D**) pathogenetic phenotypes of Col-0 and OE::*GhRDUF4D* plants at 10 and 20 dpi; (**E**,**F**) statistical analysis of the disease index and relative fungal biomass in plants in the soil. Mean ± SE, *n* = 3. “*” and “**” indicate that the data were significantly different at the *p* values of <0.05 and <0.01, respectively.

The expression level of the homologous genes *GhRDUF1A/D*, *GhRDUF4A/D*, and *GhRDUF7A/D* were upregulated after *V. dahliae* infection based on RNA-Seq data, which *GhRDUF4D* was significantly increased at 12 and 24 h after *V. dahliae* infection (Figure S8A) [11]. RT-qPCR revealed that *GhRDUF4D* expression was increased at 24 and 48 h after *V. dahliae* infection (Figure S8B). Thus, *GhRDUF4D* was selected to validate the function by overexpression in *Arabidopsis*. We observed that *GhRDUF4D* transgenic plants were more resistant to *V. dahliae* infection than the control (Figure 8A,C,D). The statistical disease index result showed that *V. dahliae* resistance in *GhRDUF4D* transgenic *Arabidopsis* was improved significantly, not only in MS medium but also in the soil (Figure 8B,E). The relative fungal biomass was reduced in *GhRDUF4D* overexpressed *Arabidopsis* (Figure 8F). These results indicate that *V. dahliae* resistance was improved by the overexpression of *GhRDUF4D* in *Arabidopsis*.

### 3.8. Knockdown of GhRDUF4D Compromise V. dahliae Resistance in Upland Cotton

**Figure 9:**
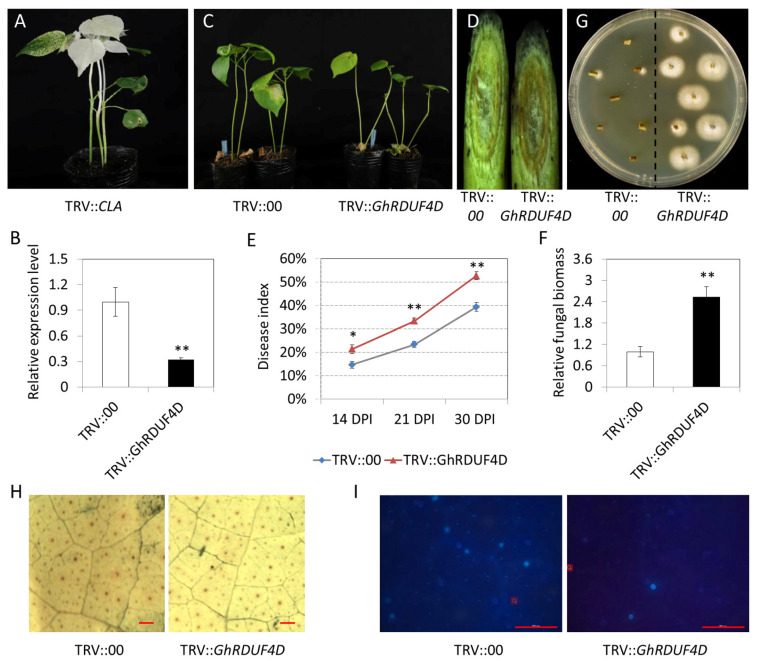
Downregulation of *GhRDUF4D* weakened upland cotton resistance to *V. dahliae*. (**A**) Photobleaching of the phenotype by inoculation TRV::*CLA*; (**B**) pathogenetic phenotypes of TRV::0 and TRV::*GhRDUF4D* cotton plants; (**C**) observation of the diagonal plane of stem vascular bundle; (**D**) the *V. dahliae* recovery assays of TRV::0 and TRV::*GhRDUF4D* stems; (**E**) relative expression level of *GhRDUF4D* in TRV::0 and TRV::*GhRDUF4D* cotton plants; (**F**) dynamic disease index of TRV::0 and TRV::*GhRDUF4D* cotton plants after Vd991 infection; (**G**) relative fungal biomass of TRV::0 and TRV::*GhRDUF4D* cotton plants; and (**H**,**I**) ROS and callose deposition observation in TRV::0 and TRV::*GhRDUF4D* cotton leaves, bar = 200μm. Mean ± SE, *n* = 3. “*” and “**” indicate that the data were significantly different at the *p* values of <0.05 and <0.01, respectively.

To investigate the biofunction of *GhRDUF4D* in the relationship between upland cotton and *V. dahliae*, the VIGS approach based on TRV was used to knockdown *GhRDUF4D* transcription levels in upland cotton. When the true leaves of the cotton seedlings were inoculated with TRV::*CLA1*, *Agrobacteria* revealed a photobleaching phenotype (Figure 9A), and the expression of *GhRDUF4D* was downregulated in TRV::*GhRDUF4D* plants (Figure 9B). The seedlings were inoculated with V991 upon reaching the three-leaf stage. The results showed that the upland cotton seedlings with *GhRDUF4D* downregulation were more susceptible to *V. dahliae* than the control (TRV::00). Indeed, *GhRDUF4D*-silenced plants displayed more wilting and yellow leaves, and a deeper vascular discoloration in stem tissues (Figure 9C,D). The quantification of the disease index in *GhRDUF4D* downregulated seedlings was higher than that of the control (Figure 9E). Fungal DNA abundance detection and fungal recovery assays displayed that greater amounts of *V. dahliae* had colonized the stem tissue of *GhRDUF4D*-silenced plants than the control (Figure 9F,G).

ROS is regarded as an indicator of disease resistance. Thus, the ROS levels of TRV::00 and TRV::*GhRDUF4D* cotton leaves were stained with DAB at 12 h after *V. dahliae* inoculation. The results showed that ROS accumulation in TRV::*GhRDUF4D* plants was significantly less than that of the TRV::00 plants (Figure 9H). Callose deposition was evaluated by aniline blue staining. Compared with the control, the density of callose depositions was reduced, with more dead cells observed in *GhRDUF4D*-silenced cotton leaves (Figure 9I). These results indicate that the reduced expression of *GhRDUF4D* compromises *V. dahliae* resistance in upland cotton, reflecting the positive role of *GhDUF4D* in plant responses to pathogenic fungal infection.

## 4. Discussion

### 4.1. Evolution and Functional Diversification of GhRDUF Genes

The RING-containing proteins ubiquitously function in light signaling, phosphate homeostasis [57], anther development [58], plant defense, and pathogen response [25,29,30,31,32,33]. We analyzed the evolution and expression of *GhRDUF* members in cotton plants to investigate the potential role of the *RDUF* genes. In addition, phylogenetic trees were constructed to determine the evolutionary relationship of RDUF proteins in plants. Gene duplication plays a major role in plant genome evolution. Based on our findings, the *GhRDUF* genes were duplicated frequently during cotton evolution. Only one *RDUF* gene was detected in each group of *Arabidopsis thaliana* and *Theobroma cacao*, however, two or three members were identified in diploid cottons (*G. arboretum* and *G. raimondii*). Of these, both the *RDUF2A/3D* and *RDUF5A/D* clades, and the *RDUF1A/D* and *RDUF4A/D* clades duplicated only once, whereas the *RDUF3A/2D*, *RDUF6A/D*, and *RDUF7A/D* clades duplicated twice during cotton evolution. Based on their chromosomal localization, the *RDUF* genes may have undergone segmental duplication in cotton evolution (Figure 3 and Figure S2). These results indicate that the *GhRDUF* genes duplicated after the cotton genus diverged from cacao.

The duplicated gene pairs possibly underwent three alternative fates during evolution, including nonfunctionalization, neofunctionalization, and subfunctionalization [56]. Thus, we investigated the expression patterns of the *GhRDUF* duplicated genes. The diverse expression patterns indicated that the *GhRDUF* genes have experienced functional diversification. We observed that the *GhRDUF2A/3D* genes were abundant during cotton growth and development, but its duplicated gene *GhRDUF5A/D* was barely expressed in the developing ovules or fibers (Figure 6). The *GhRDUF2A/3D* and *GhRDUF7A/D* genes had reduced expression levels in different cotton tissues and the developing ovules and fibers, whereas its duplicated gene *GhRDUF6A/D* had increased expression in many tissues and developing ovules. These results imply that the duplicated *GhRDUF* genes may have experienced neofunctionalization and subfunctionalization during the evolution of upland cotton.

### 4.2. RDUF Function in Plants Adapting to Abiotic Stress

RING-containing proteins are key players in plant adaptation to abiotic stress. Many U-box E3 ubiquitin ligases participate in stress management, including *TaPUB1* in salt stress [59], *CaPUB1* in cold and drought stress [60], and *AtPUB48* in thermotolerance response [61]. The RING-type E3 ligase, SDIR1, modulates the salt and drought stress responses via ABA signaling in *Arabidopsis* [29,30]. The RING-H2 type E3 ligase, OsSIRH2-14, degrades the salt-related protein OsHKT2;1 via the ubiquitin/26S proteasome and enhances salinity tolerance in rice [62]. The function of RING-DUF1117-containing proteins has rarely been reported. Expression analysis revealed that *AtRDUF1* is more abundant during cold, heat, and salt stress [63]. *AtRDUF1* positively regulates salt stress, and the suppression of *AtRDUF1* and *AtRDUF2* reduces the plant’s tolerance to ABA-mediated drought stress in *Arabidopsis* [34,35]. These results indicate that RDUF is involved in the abiotic stress response. Thus, we analyzed the potential role of GhRDUFs in upland cotton’s adaptation to abiotic stress. We detected many *cis*-elements involved in environmental adaptation in GhRDUF promoter regions: more than four ARE *cis*-elements were detected in GhRDUF3A/D, GhRDUF5A/D, and GhRDUF6A promoter regions; three STRE elements were found in GhRDUF5D and GhRDUF7A/D promoters; and three WUN-motif elements were found in GhRDUF2D and GhRDUF7A/D promoter regions (Figure 4). The TF-gene regulatory network showed that many stress-related TFs are predicted to regulate *GhRDUFs* expression, including MYB, C2H2, and Dof (Figure S7). It has been previously reported that ThMYB8 enhanced salt stress tolerance in *Tamarix hispida* and *Arabidopsis* [64], a C2H2-type ZFP gene, *PeSTZ1*, enhances freezing tolerance in *Poplar* [65], and MdDof54 promotes drought resistance in apples [66]. Thus, we investigated the expression patterns of the *GhRDUF* genes in response to cold, heat, drought, and salt stress. The *GhRDUF7A/D* homologous genes responded to cold and heat stress at 3 and 6 h, respectively, and showed increased expression under drought and salt stresses at 24 h, with three STRE elements detected in its promoter regions. In addition, the expression level of *GhRDUF3D* was upregulated at 1, 6, and 12 h when exposed to drought conditions, and two MBS elements were present in its promoter. These results suggest that the *GhRDUF* genes participate in cotton’s adaptation to abiotic stress.

### 4.3. GhRDUF4D Participates in Resistance to V. dahliae

Hormones play a vital role in plant resistance to microorganisms, including pathogenic fungi. JA, SA, and ET are the primary hormones involved in plant inner immunity [6,7,8]. Many *cis*-elements involved in hormones were detected in the GhRDUF promoter regions (Figure 4). In this study, the expression levels of many *GhRDUF* genes were increased during hormonal responses (Figure 7). The expression of the *GhRDUF2A/D* homologous genes was elevated at 3 and 6 h under MeJA conditions, and induced at 12 and 24 h under MeSA conditions. In addition, we observed a decrease in the expression of *GhRDUF4A/D*, with respect to the MeJA and MeSA responses at many time points (Figure 7). RING-containing proteins have been widely reported to play a substantial role in plant disease resistance [67]. ATL9, a RING zinc finger protein which expression levels are induced by chitin, as well as the RING domain, are important for the resistant phenotype of ATL9 [68,69]. XB3 contains a RING finger motif and displays E3 ubiquitin ligase activity. Reduced *XB3* expression is involved in resistance to *Xanthomonas oryzae* pv oryzae [32]. The overexpression of *OsPUB41*, a rice E3 ubiquitin ligase, enhanced tolerance to *Xoo* and *Rhizoctonia solani* infections in rice and *Arabidopsis* [70]. The mRNA level of RING-H2 type *BRH1* had induced rapidly by the pathogen elicitor chitin [71]. The transcript level of another RING-H2 protein, *ATL2*, was induced after incubation with pathogen elicitors [72]. OsRFPH2-10, a RING-H2 finger E3 ubiquitin ligase, plays a role in the antiviral defense of rice against the *rice dwarf virus* infection [33]. In cotton, resistance to *V. dahliae* was shown to improve by the inhibiting of the E3 ubiquitin ligase activity of GhPUB17 [73]; however, the role of RING-RDUF1117 in biotic stress remains unclear. In the present study, we observed that the gene expression levels of *GhRDUF1A/D*, *GhRDUF4A/D*, and *GhRDUF7A/D* were elevated upon *V. dahliae* infection (Figure S7). Consistently, more W-box elements were detected in the GhRDUF1A/D and GhRDUF4A/D promoter regions, particularly in the GhRDUF4D promoter (Figure 4). This result suggests that *GhRDUF4D* is involved in cotton’s defense to pathogen infection. To verify this speculation, overexpression and the VIGS approach were used to investigate the role of *GhRDUF4D* under *V. dahliae* infection. The results revealed that *Arabidopsis* plants that overexpress *GhRDUF4D* were more resistant to *V. dahliae*, and that *GhRDUF4D* downregulation in cotton plants made them more sensitive to *V. dahliae* infection, compared with the control.

miRNA regulates target genes in plant development and aids in the plant’s adaptation to the environment. In recent years, miRNAs have been characterized as key players in plants’ responses to pathogens. Because NBS-LRR proteins have been associated with effector-triggered immunity, there is a good correlation between species with large numbers of NBS-LRR genes and those with miR482 superfamily members [74,75]. This result suggests that miR482 plays an important role in plant immunity. Studies have reported that miR482 suppressed *Phytophthora* and *V. dahliae* resistance in tomatoes and potatoes [76,77,78]. In cotton, it has been reported that miR482 regulates NBS-LRR defense genes during fungal pathogen infection [79]. In the present study, *GhRDUF4D* was predicted to be regulated by two miR482 (gra-miR482b and gra-miR482c). Furthermore, gra-miR482c regulated *GhRDUF4D* at two binding sites, suggesting that *GhRDUF4D* is regulated by miR482. Hence, a transient transformation assay was performed to examine the regulatory network of *GhRDUF4D* and gra-miR482c in tobacco leaves. The fluorescent intensity and *GFP* expression level in *GhRDUF4D-GFP* and gra-miR482c lanes displayed an apparent reduction compared with the *GhRDUF4D-GFP* and gra-miR482c-mut lanes (Figure 5). These results indicate that *GhRDUF4D* might be regulated by gra-miR482c *in vivo*.

## 5. Conclusions

In conclusion, we detected and analyzed RDUF family members in cotton, and elucidated their roles in the biotic and abiotic stress responses. We found that the *GhRDUF* genes were mostly involved in the stress response, whereas the *GhRDUF4D* genes were involved in resistance to *V. dahliae* infections. The findings of this study contribute to the understanding of the *RDUF* genes in the development of resistant varieties. Moreover, *GhRDUF4D* might be a vital candidate gene applied for genetic transformation in cotton, or might be a marker gene used for *V. dahliae* resistance variety selection for cotton breeders.

## Figures and Tables

**Table 1:** Identification of the *RDUF* genes in *G. arboretum*, *G. raimondii*, *G. hirsutum*, and *G. barbadense*. Abbreviations: chlo, chloroplast; cyto, cytoplasm; mito, mitochondrion; nucl, nucleus.

Name	Gene Locus ID	Nucleic Acid			Amino Acid				
		Location	CDS (bp)	Exons	Size (aa)	Mw (Da)	pI	Formula	Subcellular Location
*GaRDUF1*	Ga01G2247.1	Chr01: 104114537-104114537	1095	1	364	40,145.64	6.72	C_1709_H_2699_N_529_O_553_S_20_	cyto: 5, nucl: 3, chlo: 2
*GaRDUF2*	Ga04G0720.1	Chr04: 15879489-15880598	1110	1	369	41,080.80	8.36	C_1780_H_2753_N_547_O_545_S_17_	nucl: 8, mito: 4, chlo: 1
*GaRDUF3*	Ga07G1079.1	Chr07: 15730492-15731585	1023	2	340	38,340.47	5.96	C_1655_H_2554_N_498_O_525_S_16_	chlo: 6, mito: 4, nucl: 2
*GaRDUF4*	Ga09G1752.1	Chr09: 75411415-75412479	1065	1	354	39,662.52	9.27	C_1720_H_2690_N_536_O_517_S_16_	nucl: 7, chlo: 3, mito: 3
*GaRDUF5*	Ga10G0274.1	Chr10: 3699993-3701000	1008	1	335	36,635.1	6.15	C_1588_H_2470_N_474_O_489_S_19_	chlo: 10, nucl: 2, cyto: 1
*GaRDUF6*	Ga13G0217.1	Chr13: 2143604-2144689	1086	1	361	39,945.89	7.03	C_1729_H_2687_N_519_O_529_S_23_	nucl: 14
*GrRDUF1*	Gorai.001G111900.1	Chr01: 13043565-13045611	1099	1	361	40,055.51	5.77	C_1728_H_2685_N_513_O_552_S_18_	chlo: 5, mito: 5, nucl: 1
*GrRDUF2*	Gorai.003G129300.1	Chr03: 38196500-38198583	1114	1	366	40,318.81	6.72	C_1716_H_2710_N_530_O_557_S_20_	nucl: 4, cyto: 3, mito: 3
*GrRDUF3*	Gorai.006G171100.1	Chr06: 43050323-43052612	1078	1	354	39,714.48	9.02	C_1718_H_2682_N_534_O_522_S_17_	nucl: 10, chlo: 2, mito: 2
*GrRDUF4*	Gorai.011G267000.1	Chr11: 59881340-59882767	953	2	313	34,156.21	8.39	C_1485_H_2298_N_448_O_453_S_15_	chlo: 9, nucl: 2, mito: 2
*GrRDUF5*	Gorai.012G071400.1	Chr12: 10539979-10541968	1011	1	332	36,698.02	7.59	C_1580_H_2471_N_491_O_488_S_17_	nucl: 8, mito: 3, chlo: 2
*GrRDUF6*	Gorai.012G105100.1	Chr12: 23596133-23598256	1123	1	369	41,122.88	8.36	C_1783_H_2759_N_547_O_545_S_17_	nucl: 8, mito: 4, chlo: 2
*GrRDUF7*	Gorai.013G021800.1	Chr13: 1532332-1533783	1102	1	362	40,120.10	8.02	C_1733_H_2701_N_527_O_529_S_23_	nucl: 14
*GhRDUF1A*	GH_A03G0566	A03: 8775627-8776721	1116	1	364	40,144.7	6.72	C_1711_H_2704_N_528_O_552_S_20_	cyto: 5, nucl: 3, chlo: 2
*GhRDUF2A*	GH_A04G1023	A04: 72823528-72824637	1132	1	369	41,114.82	8.36	C_1783_H_2751_N_547_O_545_S_17_	nucl: 9, mito: 4, chlo: 1
*GhRDUF3A*	GH_A05G3679	A05: 96863898-96864839	960	1	313	34,110.2	7.59	C_1470_H_2308_N_450_O_457_S_16_	nucl: 7, mito: 4, chlo: 2
*GhRDUF4A*	GH_A07G1092	A07: 16689279-16690247	988	1	322	36,130.2	5.25	C_1566_H_2418_N_456_O_497_S_17_	chlo: 6, mito: 4, nucl: 2
*GhRDUF5A*	GH_A09G1709	A09: 74061366-74062430	1086	1	354	39,765.64	9.25	C_1727_H_2695_N_537_O_517_S_16_	mito: 7, nucl: 3.5, chlo: 3
*GhRDUF6A*	GH_A10G2448	A10: 111936421-111937428	1028	1	335	36,635.1	6.15	C_1588_H_2470_N_474_O_489_S_19_	chlo: 10, nucl: 2, cyto: 1
*GhRDUF7A*	GH_A13G0199	A13: 2147580-2148665	1107	1	361	39,948.85	7.03	C_1727_H_2684_N_520_O_530_S_23_	nucl: 14
*GhRDUF1D*	GH_D03G1398	D03: 45757090-45758175	1107	1	361	39,809.38	6.72	C_1696_H_2687_N_525_O_546_S_20_	cyto: 4, chlo: 3, nucl: 3
*GhRDUF2D*	GH_D04G0664	D04: 11134024-11135037	1034	1	337	37,207.58	8.34	C_1602_H_2506_N_498_O_495_S_17_	nucl: 9, mito: 3, chlo: 2
*GhRDUF3D*	GH_D04G1354	D04: 44739659-44740768	1132	1	369	41,202.03	8.56	C_1788_H_2768_N_550_O_543_S_17_	nucl: 9, mito: 3, chlo: 2
*GhRDUF4D*	GH_D07G1077	D07: 13116176-13117261	1107	1	361	40,221.74	5.82	C_1737_H_2699_N_515_O_553_S_18_	chlo: 6, mito: 4, nucl: 2
*GhRDUF5D*	GH_D09G1658	D09: 43682720-43683784	1086	1	354	39,723.49	8.89	C_1720_H_2681_N_533_O_522_S_17_	nucl: 9, mito: 3, chlo: 1
*GhRDUF6D*	GH_D10G2556	D10: 64049541-64050551	1031	1	336	36,686.15	6.89	C_1590_H_2471_N_477_O_488_S_19_	chlo: 8, nucl: 3, mito: 2
*GhRDUF7D*	GH_D13G0196	D13: 1703107-1704195	1110	1	362	40,142.16	8.02	C_1735_H_2703_N_529_O_527_S_23_	nucl: 14
*GbRDUF1A*	GB_A03G0554	A03: 8482495-8483589	1116	1	364	40,144.7	6.72	C_1711_H_2704_N_528_O_552_S_20_	cyto: 5, nucl: 3, chlo: 2
*GbRDUF2A*	GB_A04G1063	A04: 67240547-67241656	1132	1	369	41,080.8	8.36	C_1780_H_2753_N_547_O_545_S_17_	nucl: 8, mito: 4, chlo: 1
*GbRDUF3A*	GB_A05G3770	A05: 94125157-94126098	960	1	313	34,134.26	7.57	C_1474_H_2312_N_448_O_457_S_16_	nucl: 6, mito: 5, chlo: 2
*GbRDUF4A*	GB_A07G1079	A07: 17016234-17017202	988	1	322	36,188.24	5.17	C_1568_H_2420_N_456_O_499_S_17_	chlo: 6, mito: 4, nucl: 2
*GbRDUF5A*	GB_A09G1832	A09: 70138025-70139089	1086	1	354	39,716.57	9.27	C_1722_H_2692_N_538_O_517_S_16_	mito: 7, nucl: 3.5, chlo: 3
*GbRDUF6A*	GB_A10G2617	A10: 108379933-108380940	1028	1	335	36,587.06	6.15	C_1584_H_2470_N_474_O_489_S_19_	chlo: 9, nucl: 2, mito: 2
*GbRDUF7A*	GB_A13G0200	A13: 2045827-2048345	1312	2	428	46,919.93	8.38	C_2038_H_3185_N_603_O_622_S_26_	nucl: 14
*GbRDUF1D*	GB_D03G1418	D03: 45690505-45691611	1129	1	368	40,430.98	6.72	C_1719_H_2726_N_532_O_559_S_20_	nucl: 4, chlo: 3, cyto: 3
*GbRDUF2D*	GB_D04G0687	D04: 10848147-10850667	1049	2	342	37,749.2	8.05	C_1625_H_2541_N_503_O_503_S_18_	nucl: 9, mito: 3, chlo: 2
*GbRDUF3D*	GB_D04G1434	D04: 45218042-45219151	1132	1	369	41,132.92	8.36	C_1785_H_2761_N_547_O_544_S_17_	nucl: 8, mito: 4, chlo: 2
*GbRDUF4D*	GB_D07G1081	D07: 13412269-13413354	1107	1	361	40,182.70	5.89	C_1734_H_2698_N_516_O_552_S_18_	chlo: 5, mito: 5, nucl: 1
*GbRDUF5D*	GB_D09G1672	D09: 45246636-45247700	1086	1	354	39,735.54	8.89	C_1722_H_2685_N_533_O_521_S_17_	nucl: 9, mito: 3, chlo: 2
*GbRDUF6D*	GB_D10G2571	D10: 63188105-63189115	1031	1	336	36,658.1	6.46	C_1589_H_2467_N_475_O_489_S_19_	chlo: 8, mito: 3, nucl: 2
*GbRDUF7D*	GB_D13G0189	D13: 1578513-1579601	1110	1	362	40,112.13	8.03	C_1734_H_2701_N_529_O_526_S_23_	nucl: 14

## Data Availability

Not applicable.

## Supplementary Materials

The following are available online at https://www.mdpi.com/article/10.3390/biom11081145/s1, Figure S1: Phylogenetic tree of RDUF protein family. At, *Arabidopsis thaliana*; Bn, *Brassica napus*; Ga, *G. arboretum*; Gr, *G. rai-mondii*; Gh, *G. hirsutum*; Gb, *G. barbadense*; Gm, *Glycine max*; Ha, *Helianthus annuus*; Os, *Oryza sativa*; Ta, *Triticum aes-tivum*; Tc, *Theobroma cacao*; Zm, *Zea mays*, Figure S2: Phylogenetic tree of RDUF family proteins in *G. arboretum*, *G. raimondii*, *G. hirsutum*, and *G. barbadense*, respectively, Figure S3: Gene location of RDUFs in *G. arboretum*, *G. raimondii*, *G. hirsutum*, and *G. barbadense*, Figure S4: The synteny relationship of *RDUF* genes in *G. hirsutum*, Figure S5: The conserved motifs identified in RDUF proteins; (A) phylogenetic tree of RDUF proteins; (B) the top ten conserved motifs in RDUF proteins; (C) logos of the ten conserved motifs in RDUF proteins, Figure S6: Amino acid sequences alignment of GhRDUF proteins. The conserved domains were circled by a rectangle with a dotted line; the eight metal ligands were highlighted by a red triangle, Figure S7: The predicted target TFs of *GhRDUF* genes. The predicted regulation TFs were identified with a yellow background in a circle, the target *GhRDUF* genes were marked with a blue background in a square. The interaction levels were displayed with different degrees, Figure S8: Expression of *GhRDUF4D* was induced upon *V**. dahliae* infection. (A) Expression patterns of *GhRDUF* genes in RNA-Seq data. (B) Expression patterns of *GhRDUF4D* by the RT-qPCR approach, Figure S9: RT-PCR and RT-qPCR analysis of transgenic Arabidopsis; (A) verification of the transgenic Arabidopsis by reverse transcription PCR. M, DNA Marker 2K plus; #1,#2,#3,#4, transgenic Arabidopsis lines; Col-0, wild type; (B) real-time quantification PCR analysis of the relative expression level of *GhRDUF4D* in single copy insertion lines. Table S1: Primers used in the present study, Table S2: Identification and nomenclature of RDUF members in other species, Table S3: Ka/Ks ratio of ortholog or paralog gene pairs, Table S4: Annotation of the conserved motifs in RDUF proteins, Table S5: cis-elements predicted in 14 GhRDUF promoter regions.

## References

[1] Li F., Fan G., Lu C., Xiao G., Zou C., Kohel R.J., Ma Z., Shang H., Ma X., Wu J. (2015). Genome sequence of cultivated Upland cotton (*Gossypium hirsutum* TM-1) provides insights into genome evolution. Nat. Biotechnol..

[2] Hu Y., Chen J., Fang L., Zhang Z., Ma W., Niu Y., Ju L., Deng J., Zhao T., Lian J. (2019). *Gossypium barbadense* and *Gossypium hirsutum* genomes provide insights into the origin and evolution of allotetraploid cotton. Nat. Genet..

[3] Huang G., Wu Z., Percy R.G., Bai M., Li Y., Frelichowski J.E., Hu J., Wang K., Yu J.Z., Zhu Y. (2020). Genome sequence of *Gossypium herbaceum* and genome updates of *Gossypium arboreum* and *Gossypium hirsutum* provide insights into cotton A-genome evolution. Nat. Genet..

[4] Yang C.L., Liang S., Wang H.Y., Han L.B., Wang F.X., Cheng H.Q., Wu X.M., Qu Z.L., Wu J.H., Xia G.X. (2015). Cotton major latex protein 28 functions as a positive regulator of the ethylene responsive factor 6 in defense against *Verticillium dahliae*. Mol. Plant.

[5] Hu Q., Zhu L., Zhang X., Guan Q., Xiao S., Min L., Zhang X. (2018). GhCPK33 negatively regulates defense against *Verticillium dahliae* by phosphorylating GhOPR3. Plant Physiol..

[6] Shaban M., Miao Y., Ullah A., Khan A.Q., Menghwar H., Khan A.H., Ahmed M.M., Tabassum M.A., Zhu L. (2018). Physiological and molecular mechanism of defense in cotton against *Verticillium dahliae*. Plant Physiol. Biochem..

[7] Zhang J., Hu H.L., Wang X.N., Yang Y.H., Zhang C.J., Zhu H.Q., Shi L., Tang C.M., Zhao M.W. (2020). Dynamic infection of *Verticillium dahliae* in upland cotton. Plant Biol..

[8] Long L., Xu F.C., Zhao J.R., Li B., Xu L., Gao W. (2020). *GbMPK3* overexpression increases cotton sensitivity to *Verticillium dahliae* by regulating salicylic acid signaling. Plant Sci..

[9] Liu N., Sun Y., Pei Y., Zhang X., Wang P., Li X., Li F., Hou Y. (2018). A pectin methylesterase inhibitor enhances resistance to *Verticillium* wilt. Plant Physiol..

[10] Zhang Y., Wu L., Wang X., Chen B., Zhao J., Cui J., Li Z., Yang J., Wu L., Wu J. (2019). The cotton laccase gene GhLAC15 enhances *Verticillium* wilt resistance via an increase in defence-induced lignification and lignin components in the cell walls of plants. Mol. Plant Pathol..

[11] Zhang Y., Jin Y., Gong Q., Li Z., Zhao L., Han X., Zhou J., Li F., Yang Z. (2019). Mechanismal analysis of resistance to *Verticillium dahliae* in upland cotton conferred by overexpression of *RPL18A-6* (*Ribosomal Protein L18A-6*). Ind. Crop. Prod..

[12] Zhou Y., Sun L., Wassan G.M., He X., Shaban M., Zhang L., Zhu L., Zhang X. (2019). GbSOBIR1 confers *Verticillium* wilt resistance by phosphorylating the transcriptional factor GbbHLH171 in *Gossypium barbadense*. Plant Biotechnol. J..

[13] Chen B., Zhang Y., Yang J., Zhang M., Ma Q., Wang X., Ma Z. (2020). The G-protein a subunit GhGPA positively regulates *Gossypium hirsutum* resistance to *Verticillium dahliae* via induction of SA and JA signaling pathways and ROS accumulation. Crop J..

[14] Smalle J., Vierstra R.D. (2004). The ubiquitin 26S proteasome proteolytic pathway. Annu. Rev. Plant Biol..

[15] Kraft E., Stone S.L., Ma L., Su N., Gao Y., Lau O.S., Deng X.W., Callis J. (2005). Genome analysis and functional characterization of the E2 and RING-type E3 ligase ubiquitination enzymes of *Arabidopsis*. Plant Physiol..

[16] Glickman M.H., Ciechanover A. (2002). The ubiquitin-proteasome proteolytic pathway: Destruction for the sake of construction. Physiol. Rev..

[17] Dye B.T., Schulman B.A. (2007). Structural mechanisms underlying posttranslational modification by ubiquitin-like proteins. Annu. Rev. Biophys. Biomol. Struct..

[18] Hunter T. (2007). The age of crosstalk: Phosphorylation, ubiquitination, and beyond. Mol. Cell.

[19] Kosarev P., Mayer K.F., Hardtke C.S. (2002). Evaluation and classification of RING-finger domains encoded by the Arabidopsis genome. Genome Biol..

[20] Freemont P.S. (1993). The RING finger: A novel protein sequence motif related to the zinc finger. Ann. N. Y. Acad. Sci..

[21] Callis J. (2014). The ubiquitination machinery of the ubiquitin system. Arabidopsis B.

[22] Lorick K.L., Jensen J.P., Fang S., Ong A.M., Hatakeyama S., Weissman A.M. (1999). RING fingers mediate ubiquitin-conjugating enzyme (E2)-dependent ubiquitination. Proc. Natl. Acad. Sci. USA.

[23] Freemont P.S., Hanson I.M., Trowsdale J. (1991). A novel cysteine-rich sequence motif. Cell.

[24] Lovering R., Hanson I.M., Borden K.L., Martin S., O’Reilly N.J., Evan G.I., Rahman D., Pappin D.J., Trowsdale J., Freemont P.S. (1993). Identification and preliminary characterization of a protein motif related to the zinc finger. Proc. Natl. Acad. Sci. USA.

[25] Stone S.L., Hauksdóttir H., Troy A., Herschleb J., Kraft E., Callis J. (2005). Functional analysis of the RING-type ubiquitin ligase family of Arabidopsis. Plant Physiol..

[26] Wang D., Guo Y., Wu C., Yang G., Li Y., Zheng C. (2008). Genome-wide analysis of CCCH zinc finger family in Arabidopsis and rice. BMC Genom..

[27] Gao Y., Li M.Y., Zhao J., Zhang Y.C., Xie Q.J., Chen D.H. (2016). Genome-wide analysis of RING finger proteins in the smallest free-living photosynthetic eukaryote *Ostreococus tauri*. Mar. Genomics.

[28] Alam I., Yang Y.Q., Wang Y., Zhu M.L., Wang H.B., Chalhoub B., Lu Y.H. (2017). Genome-wide identification, evolution and expression analysis of RING finger protein genes in *Brassica rapa*. Sci. Rep..

[29] Zhang Y., Yang C., Li Y., Zheng N., Chen H., Zhao Q., Gao T., Guo H., Xie Q. (2007). SDIR1 is a RING finger E3 ligase that positively regulates stress-responsive abscisic acid signaling in Arabidopsis. Plant Cell.

[30] Zhang H., Cui F., Wu Y., Lou L., Liu L., Tian M., Ning Y., Shu K., Tang S., Xie Q. (2015). The RING finger ubiquitin E3 ligase SDIR1 targets SDIR1-INTERACTING PROTEIN1 for degradation to modulate the salt stress response and ABA signaling in Arabidopsis. Plant Cell.

[31] Cheung M.Y., Zeng N.Y., Tong S.W., Li F.W., Zhao K.J., Zhang Q., Sun S.S., Lam H.M. (2007). Expression of a RING-HC protein from rice improves resistance to *Pseudomonas syringae* pv. tomato DC3000 in transgenic *Arabidopsis thaliana*. J. Exp. Bot..

[32] Wang Y.S., Pi L.Y., Chen X., Chakrabarty P.K., Jiang J., De Leon A.L., Liu G.Z., Li L., Benny U., Oard J. (2006). Rice XA21 binding protein 3 is a ubiquitin ligase required for full Xa21-mediated disease resistance. Plant Cell.

[33] Liu L., Jin L., Huang X., Geng Y., Li F., Qin Q., Wang R., Ji S., Zhao S., Xie Q.I. (2014). OsRFPH2-10, a ring-H2 finger E3 ubiquitin ligase, is involved in rice antiviral defense in the early stages of *rice dwarf virus* infection. Mol. Plant.

[34] Li J., Han Y., Zhao Q., Li C., Xie Q., Chong K., Xu Y. (2013). The E3 ligase AtRDUF1 positively regulates salt stress responses in *Arabidopsis thaliana*. PLoS ONE.

[35] Kim S.J., Ryu M.Y., Kim W.T. (2012). Suppression of Arabidopsis RING-DUF1117 E3 ubiquitin ligases, AtRDUF1 and AtRDUF2, reduces tolerance to ABA-mediated drought stress. Biochem. Biophys. Res. Commun..

[36] Libault M., Wan J., Czechowski T., Udvardi M., Stacey G. (2007). Identification of 118 Arabidopsis transcription factor and 30 ubiquitin-ligase genes responding to chitin, a plant-defense elicitor. Mol. Plant Microbe Interact..

[37] Du X., Huang G., He S., Yang Z., Sun G., Ma X., Li N., Zhang X., Sun J., Liu M. (2018). Resequencing of 243 diploid cotton accessions based on an updated A genome identifies the genetic basis of key agronomic traits. Nat. Genet..

[38] Paterson A.H., Wendel J.F., Gundlach H., Guo H., Jenkins J., Jin D., Llewellyn D., Showmaker K.C., Shu S., Udall J. (2012). Repeated polyploidization of *Gossypium* genomes and the evolution of spinnable cotton fibres. Nature.

[39] Yu J., Jung S., Cheng C.H., Ficklin S.P., Lee T., Zheng P., Jones D., Percy R.G., Main D. (2014). CottonGen: A genomics, genetics and breeding database for cotton research. Nucleic Acids Res..

[40] Gasteiger E., Hoogland C., Gattiker A., Duvaud S., Wilkins M.R., Appel R.D., Bairoch A., Walker J.M. (2005). Protein Identification and Analysis Tools on the ExPASy server. The Proteomics Protocols Handbook.

[41] Lescot M., Déhais P., Thijs G., Marchal K., Moreau Y., Van de Peer Y., Rouzé P., Rombauts S. (2002). PlantCARE, a database of plant cis-acting regulatory elements and a portal to tools for in silico analysis of promoter sequences. Nucleic Acids Res..

[42] Kumar S., Stecher G., Li M., Knyaz C., Tamura K. (2018). MEGA X: Molecular evolutionary genetics analysis across computing platforms. Mol. Biol. Evol..

[43] Letunic I., Bork P. (2019). Interactive Tree of Life (iTOL) v4: Recent updates and new developments. Nucleic Acids Res..

[44] Hu B., Jin J., Guo A.Y., Zhang H., Luo J., Gao G. (2015). GSDS 2.0: An upgraded gene feature visualization server. Bioinformatics.

[45] Letunic I., Bork P. (2018). 20 years of the SMART protein domain annotation resource. Nucleic Acids Res..

[46] Chen C., Chen H., Zhang Y., Thomas H.R., Frank M.H., He Y., Xia R. (2020). TBtools: An integrative toolkit developed for interactive analyses of big biological data. Mol. Plant.

[47] Bailey T.L., Boden M., Buske F.A., Frith M., Grant C.E., Clementi L., Ren J., Li W.W., Noble W.S. (2009). MEME SUITE: Tools for motif discovery and searching. Nucleic Acids Res..

[48] Voorrips R.E. (2002). MapChart: Software for the graphical presentation of linkage maps and QTLs. J. Hered..

[49] Wang Y., Tang H., Debarry J.D., Tan X., Li J., Wang X., Lee T.H., Jin H., Marler B., Guo H. (2012). MCScanX: A toolkit for detection and evolutionary analysis of gene synteny and collinearity. Nucleic Acids Res..

[50] Krzywinski M., Schein J., Birol I., Connors J., Gascoyne R., Horsman D., Jones S.J., Marra M.A. (2009). Circos: An information aesthetic for comparative genomics. Genome Res..

[51] Pertea M., Kim D., Pertea G.M., Leek J.T., Salzberg S.L. (2016). Transcript-level expression analysis of RNA-seq experiments with HISAT, StringTie and Ballgown. Nat. Protoc..

[52] Tian F., Yang D.C., Meng Y.Q., Jin J., Gao G. (2020). PlantRegMap: Charting functional regulatory maps in plants. Nucleic Acids Res..

[53] Dai X., Zhuang Z., Zhao P.X. (2018). psRNATarget: A plant small RNA target analysis server (2017 release). Nucleic Acids Res..

[54] Shannon P., Markiel A., Ozier O., Baliga N.S., Wang J.T., Ramage D., Amin N., Schwikowski B., Ideker T. (2003). Cytoscape: A software environment for integrated models of biomolecular interaction networks. Genome Res..

[55] Liu S., Sun R., Zhang X., Feng Z., Wei F., Zhao L., Zhang Y., Zhu L., Feng H., Zhu H. (2020). Genome-wide analysis of OPR family genes in cotton identified a role for *GhOPR9* in *Verticillium dahliae* resistance. Genes.

[56] Zhao Y., Guo A., Wang Y., Hua J. (2019). Evolution of PEPC gene family in *Gossypium* reveals functional diversification and *GhPEPC* genes responding to abiotic stresses. Gene.

[57] Ruan W., Guo M., Wang X., Guo Z., Xu Z., Xu L., Zhao H., Sun H., Yan C., Yi K. (2019). Two RING-finger ubiquitin E3 ligases regulate the degradation of SPX4, an internal phosphate sensor, for phosphate homeostasis and signaling in rice. Mol. Plant.

[58] Cao H., Li X., Wang Z., Ding M., Sun Y., Dong F., Chen F., Liu L., Doughty J., Li Y. (2015). Histone H2B monoubiquitination mediated by HISTONE MONOUBIQUITINATION1 and HISTONE MONOUBIQUITINATION2 is involved in anther development by regulating tapetum degradation-related genes in rice. Plant Physiol..

[59] Wang W., Wang W., Wu Y., Li Q., Zhang G., Shi R., Yang J., Wang Y., Wang W. (2020). The involvement of wheat U-box E3 ubiquitin ligase TaPUB1 in salt stress tolerance. J. Integr. Plant Biol..

[60] Min H.J., Jung Y.J., Kang B.G., Kim W.T. (2016). CaPUB1, a hot pepper U-box E3 ubiquitin ligase, confers enhanced cold stress tolerance and decreased drought stress tolerance in transgenic rice (*Oryza sativa* L.). Mol. Cells.

[61] Peng L., Wan X., Huang K., Pei L., Xiong J., Li X., Wang J. (2019). AtPUB48 E3 ligase plays a crucial role in the thermotolerance of Arabidopsis. Biochem. Biophys. Res. Commun..

[62] Park Y.C., Lim S.D., Moon J.C., Jang C.S. (2019). A rice really interesting new gene H2-type E3 ligase, OsSIRH2-14, enhances salinity tolerance via ubiquitin/26S proteasome-mediated degradation of salt-related proteins. Plant Cell Environ..

[63] Inzé A., Vanderauwera S., Hoeberichts F.A., Vandorpe M., Van Gaever T., Van Breusegem F. (2012). A subcellular localization compendium of hydrogen peroxide-induced proteins. Plant Cell Environ..

[64] Liu Z.Y., Li X.P., Zhang T.Q., Wang Y.Y., Wang C., Gao C.Q. (2021). Overexpression of *ThMYB8* mediates salt stress tolerance by directly activating stress-responsive gene expression. Plant Sci..

[65] He F., Li H.G., Wang J.J., Su Y., Wang H.L., Feng C.H., Yang Y., Niu M.X., Liu C., Yin W. (2019). PeSTZ1, a C2H2-type zinc finger transcription factor from *Populus euphratica*, enhances freezing tolerance through modulation of ROS scavenging by directly regulating PeAPX2. Plant Biotechnol. J..

[66] Chen P., Yan M., Li L., He J., Zhou S., Li Z., Niu C., Bao C., Zhi F., Ma F. (2020). The apple DNA-binding one zinc-finger protein MdDof54 promotes drought resistance. Hortic. Res..

[67] Ning Y., Wang R., Shi X., Zhou X., Wang G.L. (2016). A layered defense strategy mediated by rice E3 ubiquitin ligases against diverse pathogens. Mol. Plant.

[68] Berrocal-Lobo M., Stone S., Yang X., Antico J., Callis J., Ramonell K.M., Somerville S. (2010). ATL9, a RING zinc finger protein with E3 ubiquitin ligase activity implicated in chitin- and NADPH oxidase-mediated defense responses. PLoS ONE.

[69] Deng F., Guo T., Lefebvre M., Scaglione S., Antico C.J., Jing T., Yang X., Shan W., Ramonell K.M. (2017). Expression and regulation of ATL9, an E3 ubiquitin ligase involved in plant defense. PLoS ONE.

[70] Kachewar N.R., Gupta V., Ranjan A., Patel H.K., Sonti R.V. (2019). Overexpression of *OsPUB41*, a Rice E3 ubiquitin ligase induced by cell wall degrading enzymes, enhances immune responses in rice and Arabidopsis. BMC Plant Biol..

[71] Molnár G., Bancoş S., Nagy F., Szekeres M. (2002). Characterisation of *BRH1*, a brassinosteroid-responsive RING-H2 gene from *Arabidopsis thaliana*. Planta.

[72] Serrano M., Guzmán P. (2004). Isolation and gene expression analysis of *Arabidopsis thaliana* mutants with constitutive expression of *ATL2*, an early elicitor-response RING-H2 zinc-finger gene. Genetics.

[73] Qin T., Liu S., Zhang Z., Sun L., He X., Lindsey K., Zhu L., Zhang X. (2019). GhCyP3 improves the resistance of cotton to *Verticillium dahliae* by inhibiting the E3 ubiquitin ligase activity of GhPUB17. Plant Mol. Biol..

[74] González V.M., Müller S., Baulcombe D., Puigdomènech P. (2015). Evolution of NBS-LRR gene copies among dicot plants and its regulation by members of the miR482/2118 superfamily of miRNAs. Mol. Plant.

[75] Canto-Pastor A., Santos B.A.M.C., Valli A.A., Summers W., Schornack S., Baulcombe D.C. (2019). Enhanced resistance to bacterial and oomycete pathogens by short tandem target mimic RNAs in tomato. Proc. Natl. Acad. Sci. USA.

[76] Yang L., Mu X., Liu C., Cai J., Shi K., Zhu W., Yang Q. (2015). Overexpression of potato miR482e enhanced plant sensitivity to *Verticillium dahliae* infection. J. Integr. Plant Biol..

[77] de Vries S., Kukuk A., von Dahlen J.K., Schnake A., Kloesges T., Rose L.E. (2018). Expression profiling across wild and cultivated tomatoes supports the relevance of early miR482/2118 suppression for *Phytophthora* resistance. Proc. Biol. Sci..

[78] Jiang N., Meng J., Cui J., Sun G., Luan Y. (2018). Function identification of miR482b, a negative regulator during tomato resistance to *Phytophthora infestans*. Hortic. Res..

[79] Zhu Q.H., Fan L., Liu Y., Xu H., Llewellyn D., Wilson I. (2013). miR482 regulation of *NBS-LRR* defense genes during fungal pathogen infection in cotton. PLoS ONE.

