# Supplementary material for: Evolutionary and Characteristic Analysis of RING-DUF1117 E3 Ubiquitin Ligase Genes in Gossypium Discerning the Role of GhRDUF4D in Verticillium dahliae Resistance
Source: Biomolecules. 2021 Aug 3;11(8):1145. doi: 10.3390/biom11081145 (PMC8392396; doi:10.3390/biom11081145)
Supplement: Supplementary file 1 [file biomolecules-11-01145-s001.zip › biomolecules-1296323-supplementary/Supplementary Files/supplementary-Figures.pdf]

Supplementary Figures

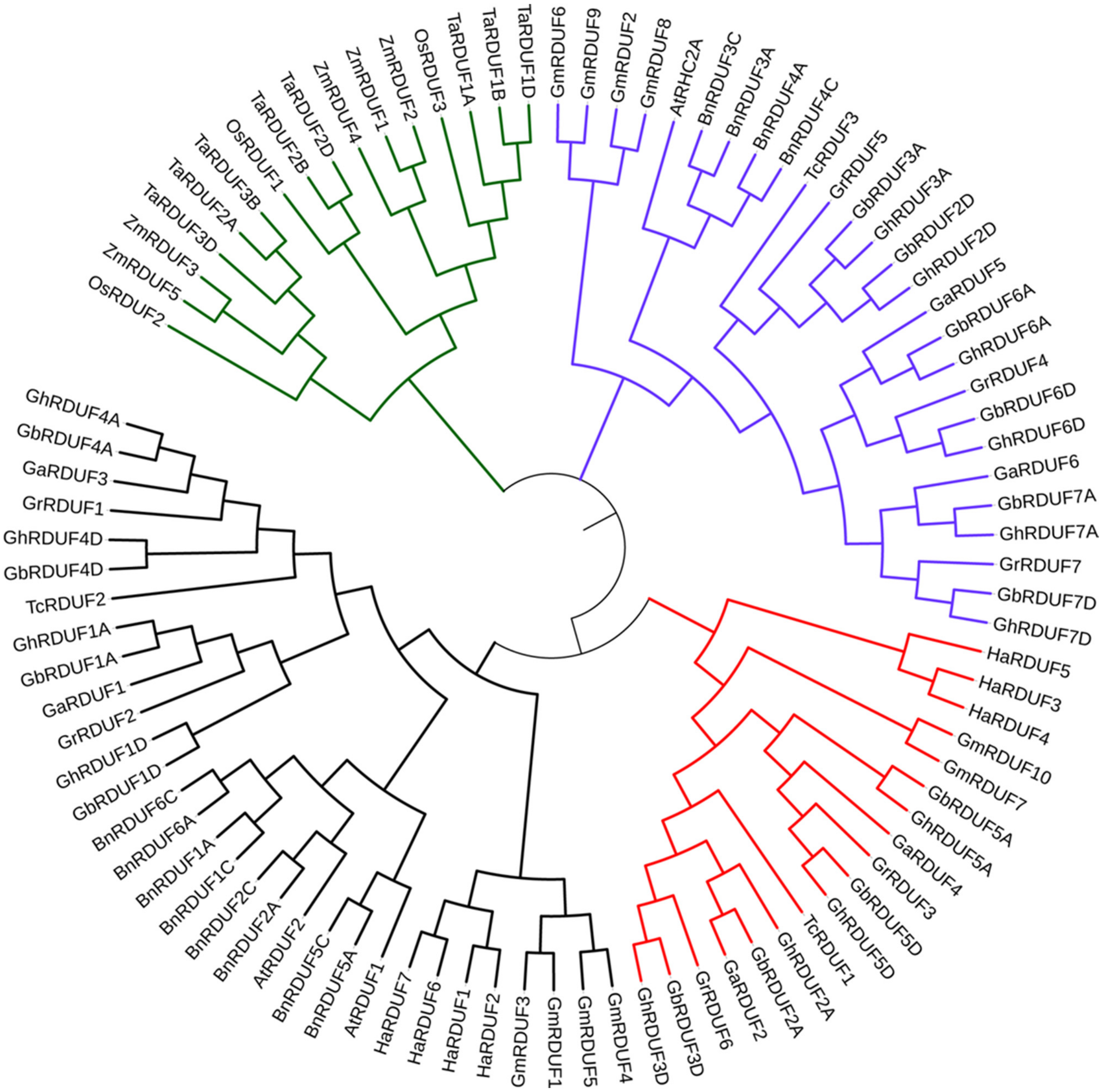

Figure S1. Phylogenetic tree of RDUF protein family. At, *Arabidopsis thaliana*; Bn, *Brassica napus*; Ga, *G. arboretum*; Gr, *G. raimondii*; Gh, *G. hirsutum*; Gb, *G. barbadense*; Gm, *Glycine max*; Ha, *Helianthus annuus*; Os, *Oryza sativa*; Ta, *Triticum aestivum*; Tc, *Theobroma cacao*; Zm, *Zea mays*.

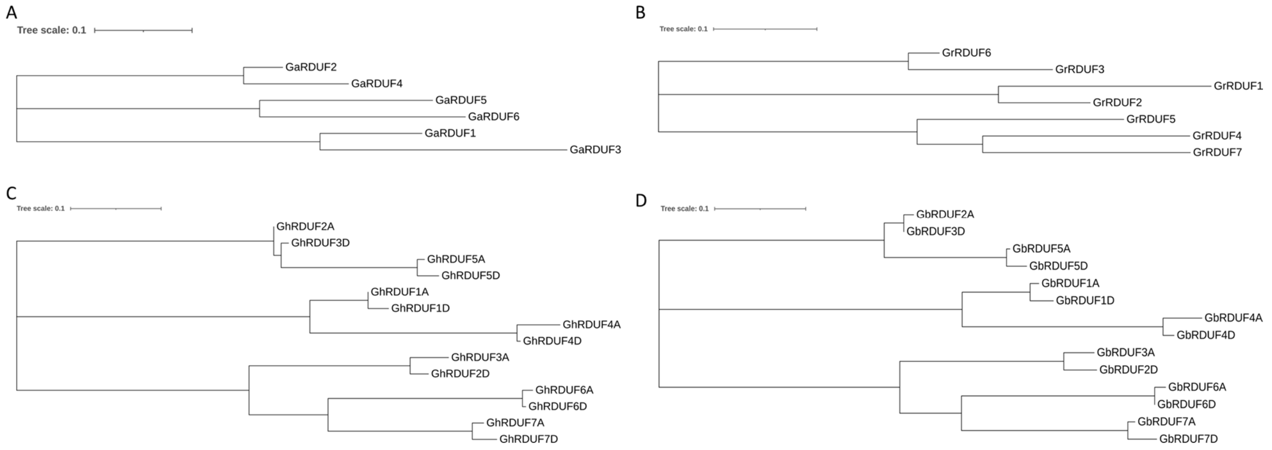

Figure S2. Phylogenetic tree of RDUF family proteins in *G. arboretum*, *G. raimondii*, *G. hirsutum* and *G. barbadense*, respectively.

Figure S3. Gene location of *RDUFs* in *G. arboretum*, *G. raimondii*, *G. hirsutum* and *G. barbadense*.

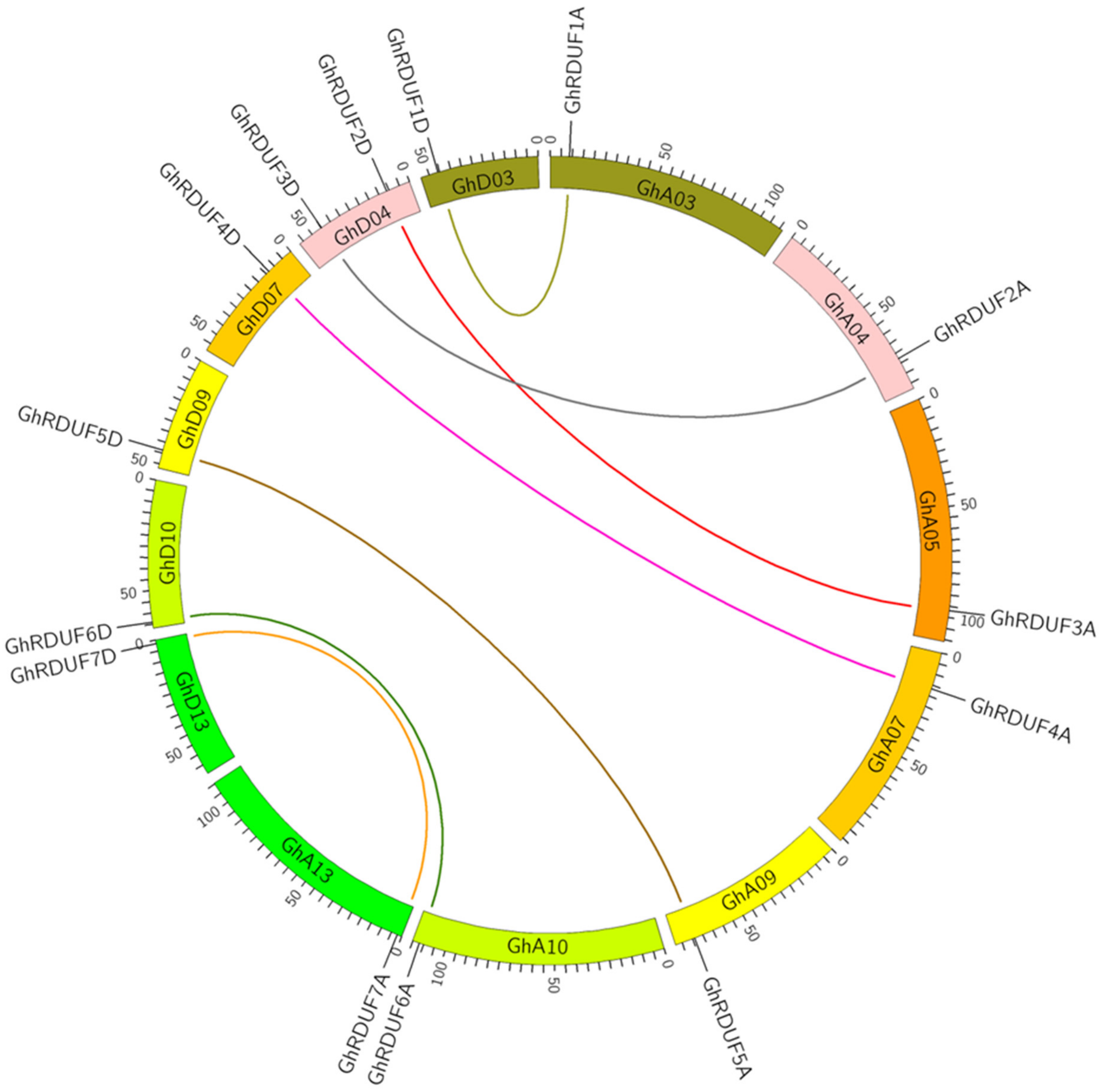

Figure S4. The synteny relationship of *RDUF* genes in *G. hirsutum*.

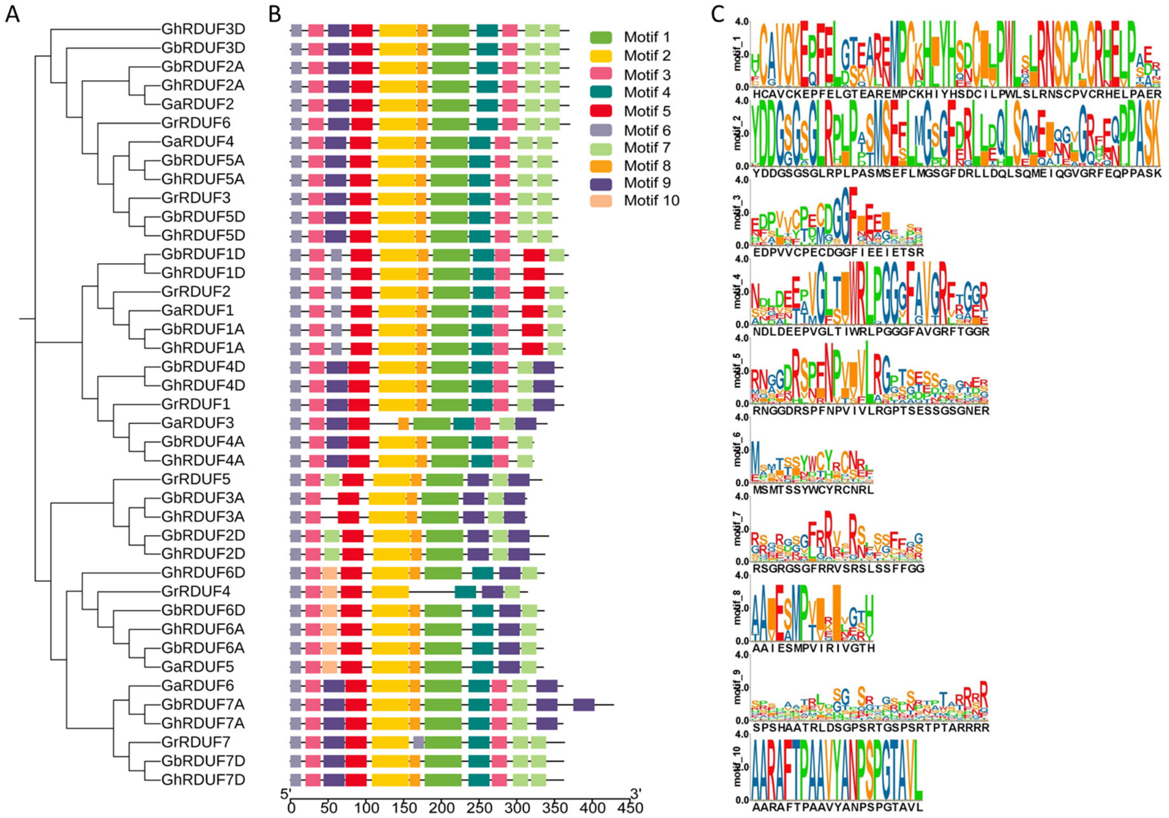

Figure S5. The conserved motifs identified in RDUF proteins. (A) Phylogenetic tree of RDUF proteins. (B) The top ten conserved motifs in RDUF proteins. (C) Logos of the ten conserved motifs in RDUF proteins.

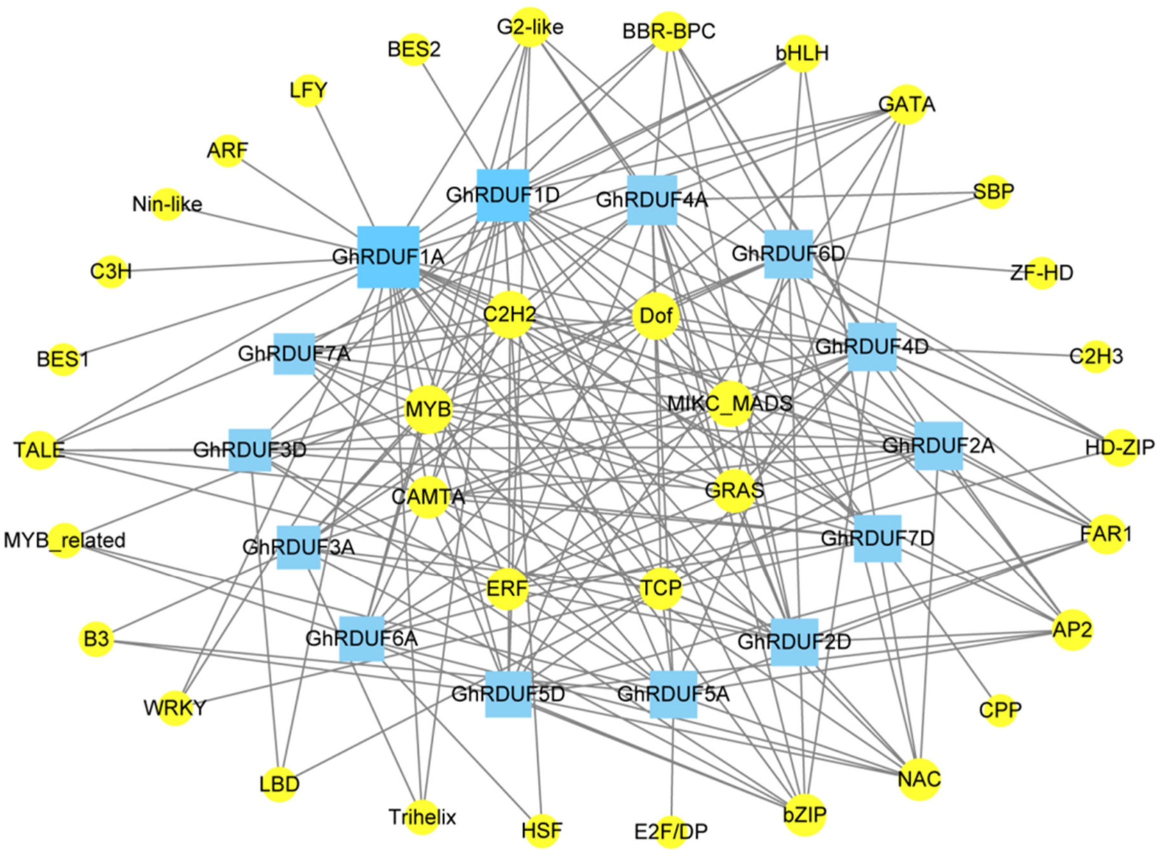

Figure S6. Amino acid sequences alignment of GhRDUF proteins. The conserved domains were circled by rectangle in dotted line; the eight metal ligands were highlighted by red triangle.

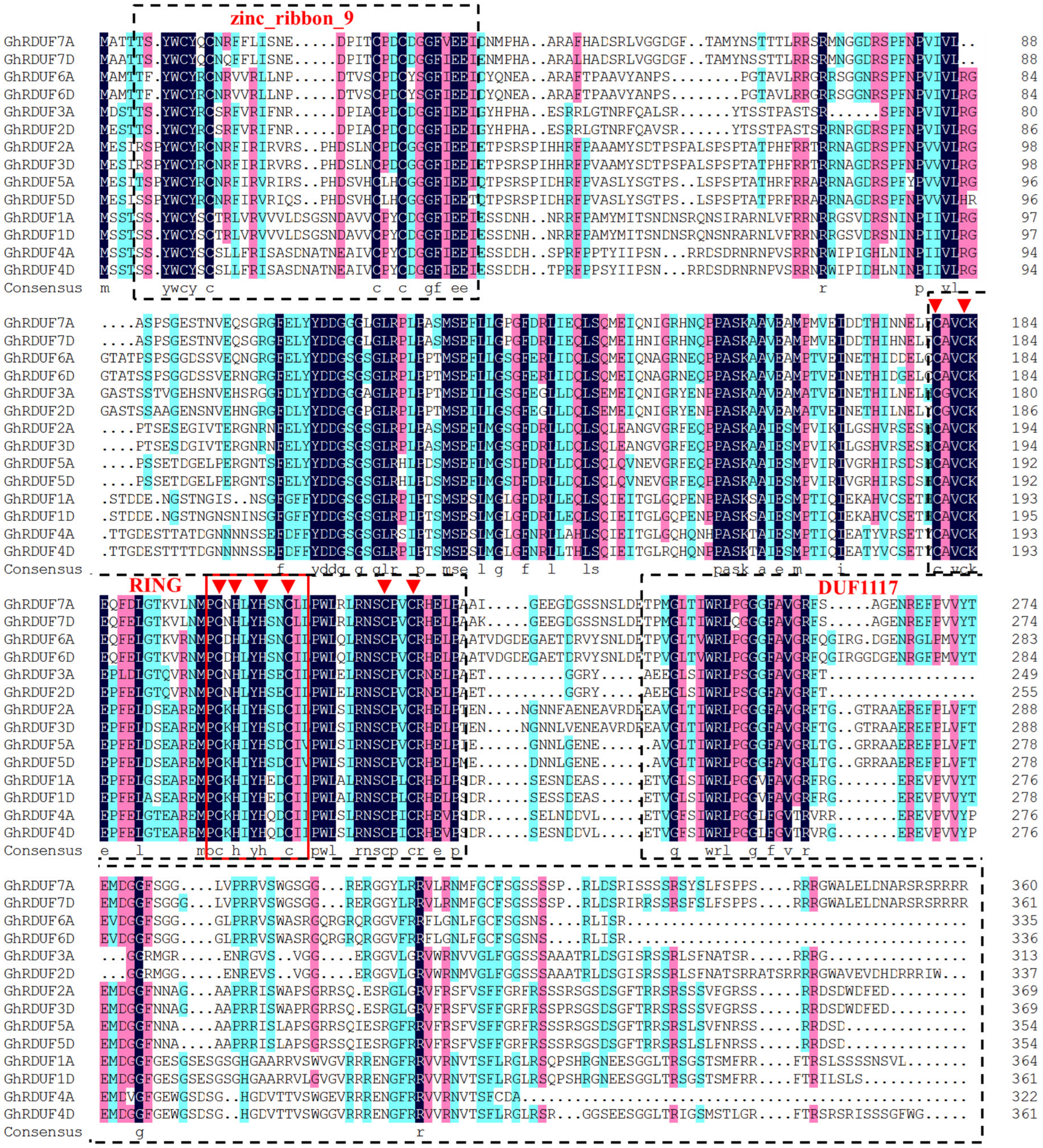

Figure S7. The predicted target TFs of *GhRDUF* genes. The predicted regulation TFs were descripted in yellow background in round, the target *GhRDUF* genes were marked with blue background in square. The interaction levels were displayed with different degrees.

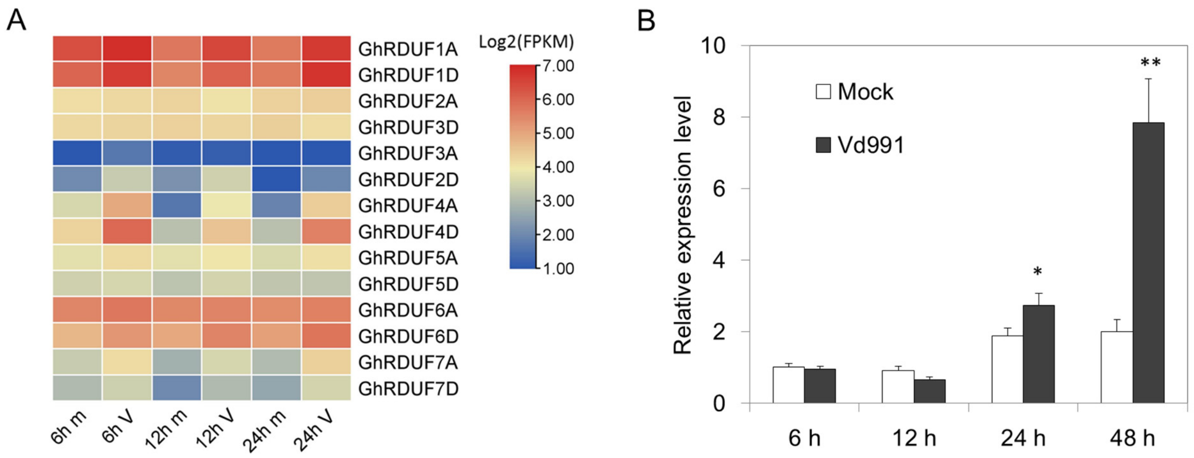

Figure S8. Expression of *GhRDUF4D* was induced upon *Verticillium dahliae* infection. (A) Expression patterns of *GhRDUF* genes in RNA-Seq data. (B) Expression patterns of *GhRDUF4D* by RT-qPCR approach.

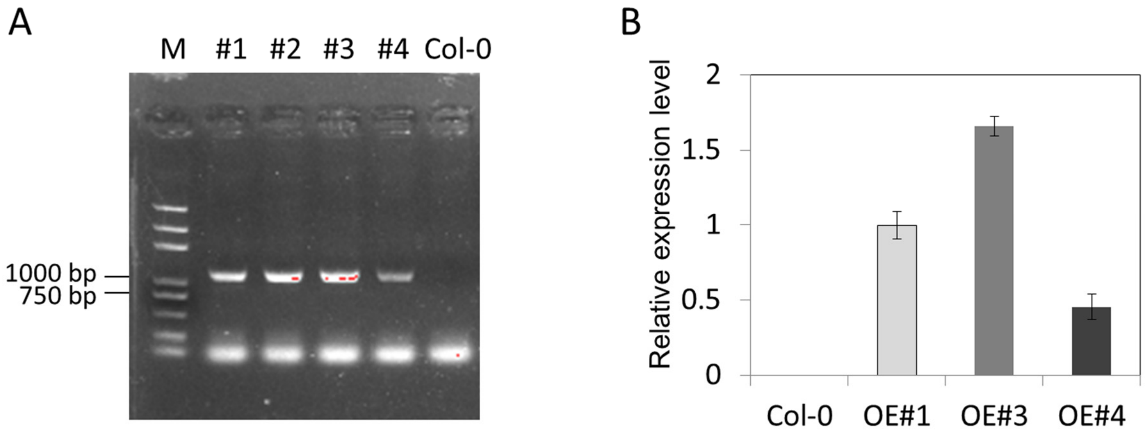

Figure S9. RT-PCR and RT-qPCR analysis of transgenic Arabidopsis. (A) Verification of the transgenic Arabidopsis by reverse transcription PCR. M, DNA Marker 2K plus; #1,#2,#3,#4, transgenic Arabidopsis lines; Col-0, wild type. (B) Real-time quantification PCR analysis of the relative expression level of *GhRDUF4D* in single copy insertion lines.
